# The Rho guanine-nucleotide exchange factor P-Rex2 exhibits structural and regulatory features distinct from the related RhoGEF P-Rex1

**DOI:** 10.1016/j.jbc.2026.113229

**Published:** 2026-06-04

**Authors:** Lauren K. Anderson, Rohan Marde, Grace Muma, Veda Nayak, Chi Phan, Sheng Li, Jennifer N. Cash

**Affiliations:** 1 Department of Molecular and Cellular Biology, University of California – Davis, Davis, California, USA; 2 Department of Medicine, University of California – San Diego, La Jolla, California, USA

**Keywords:** RhoGEF, GTPase, cryo-EM, P-Rex, Rac, inositol phosphate

## Abstract

Rho guanine-nucleotide exchange factors (RhoGEFs) activate small GTPases to drive cytoskeletal rearrangement, cell motility, and proliferation. The phosphatidylinositol-3,4,5-trisphosphate (PIP_3_)-dependent Rac exchanger (P-Rex) subfamily of RhoGEFs includes P-Rex1 and P-Rex2 which, when misregulated, contribute to cancer progression and metastasis. P-Rex activity is controlled by accessory domains that maintain the protein in a cytosolic, autoinhibited state until activated by the lipid PIP_3_ and G protein βγ subunits. While P-Rex1 autoinhibition has been structurally and biochemically characterized, P-Rex2 has remained largely unexplored. Furthermore, despite high sequence similarity and domain conservation, P-Rex homologs differ in substrate specificity and regulatory interactions, and the molecular basis for these divergences is unknown. Here, we have taken an integrative structural biology approach to investigate these gaps. Using cryo-EM, we determined the first structure of full-length P-Rex2 to moderate resolution, revealing that, while the overall structure closely resembles that of P-Rex1, there is a substantial repositioning of the N-terminal module relative to the C-terminal core. This may play a key role in precluding the intramolecular interactions between the N- and C-terminal domains that are observed in autoinhibited P-Rex1. Hydrogen-deuterium exchange mass spectrometry revealed that, unlike P-Rex1, P-Rex2 dynamics are unaffected by IP_4_, the headgroup of PIP_3_. SEC-SAXS data support that the N-terminal module itself is less dynamic, and biochemical assays show that P-Rex2 may be differently regulated by autoinhibition, likely through a mechanism divergent from P-Rex1. These findings uncover unique features in the molecular mechanisms of P-Rex2 regulation.

Rho guanine-nucleotide exchange factors (RhoGEFs) accelerate the exchange of GDP for GTP on small Rho GTPases like Rac and Cdc42 to direct actin cytoskeleton rearrangement (1, 2). This activity allows them to drive cell migration and invasion which, when misregulated, can contribute to various diseases including cancer, resulting in metastasis (3). Members of the Dbl family of RhoGEFs, which contains over 70 members, share a conserved Dbl homology (DH) catalytic domain followed by a pleckstrin homology (PH) domain that together form the catalytic core (4). Although the DH/PH tandem is well-characterized structurally (5, 6, 7), the variety of accessory domains that accompany the catalytic core across and within different RhoGEFs introduces complicated, and comparatively vastly understudied, regulatory controls on GEF activity. These include regulation through autoinhibition, membrane localization, and protein–protein interactions (1, 8). As a result, the complex molecular mechanisms governing the activity of each of these multidomain proteins are poorly understood.

The phosphatidylinositol (3,4,5)-trisphosphate (PIP_3_)-dependent Rac exchanger (P-Rex) subfamily of Dbl RhoGEFs includes two members, P-Rex1 and P-Rex2. P-Rex1 is abundantly expressed in leukocytes and neurons, whereas P-Rex2 is strongly expressed in brain, lung and liver cells (9, 10, 11, 12). *PREX2* additionally gives rise to a splice variant, P-Rex2b, which is mainly expressed in heart tissue and retains all identifiable domains except the IP4P domain, which is truncated and ends with a short alternative sequence (13). Misregulation of P-Rex family proteins is associated with varied pathologies. While P-Rex1-driven diseases, like prostate and breast cancers, are linked to overexpression (14, 15, 16), P-Rex2-driven diseases are propelled by mutation. For example, more than 23% of human hepatocellular carcinoma and 38% of squamous cell carcinoma samples harbor non-silent somatic mutations in P-Rex2 (17, 18). Additionally, mutations in P-Rex2 can lead to metastatic melanoma (19) and pancreatic cancers (20) or disrupt PI3K signaling pathways to drive diabetes and insulin resistance (21). In contrast, single and double knock-out of *PREX1* and *PREX2* genes in mouse models produces healthy, viable mice with very mild neutrophilia and a characteristic pigmentation phenotype (12, 22). Collectively, these data suggest that P-Rex is an excellent therapeutic target. Thus, characterizing the molecular mechanisms involved in P-Rex1 and P-Rex2 regulation and defining the differences between the two are important for guiding efforts to rationally design isoform-specific P-Rex modulators, both for use as tools in understanding RhoGEF regulation and for potential treatment of diseases.

P-Rex1 and P-Rex2 show 56% sequence identity and 68% sequence similarity overall, and both proteins share a conserved domain architecture consisting of an N-terminal DH/PH tandem followed by two Dishevelled, Egl-10 and Pleckstrin (DEP) domains, two Postsynaptic density protein 95, Discs large protein, and Zonula occludens-1 (PDZ) domains, and a C-terminal inositol polyphosphate-4-phosphatase-like (IP4P) domain (Fig. 1*A*). The P-Rex1 N-terminus forms a multi-domain module composed of the DH, PH, and DEP1 domains, which is flexibly tethered to the rest of the protein (Fig. 1*B*). The DH domain, which engages GTPases directly, is highly conserved as are the other domains within the N-terminal module (Fig. 1*A*). P-Rex1 also contains a C-terminal “core” composed of the tightly associated DEP2, PDZ1, PDZ2, and IP4P domains, which exhibit the highest sequence divergence between the two proteins (Fig. 1, *A* and *B*). Furthermore, a flexibly tethered subdomain of ∼250 residues extends out from the IP4P domain and forms a 4-helix bundle (4HB).

**Figure 1:**
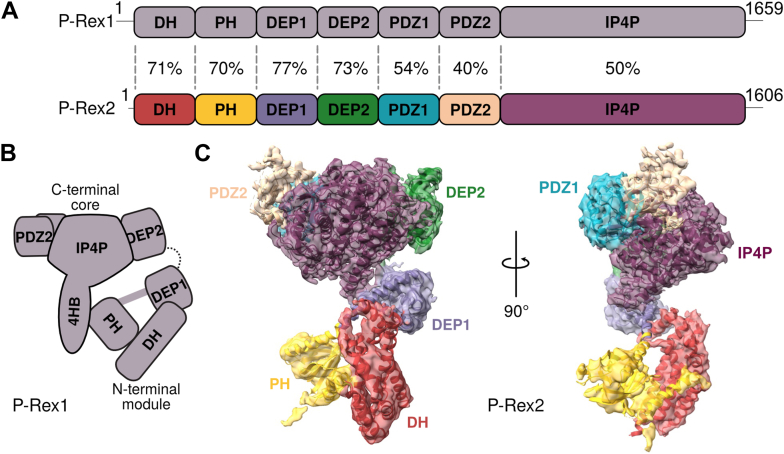
**P-Rex2 exhibits a structure that is overall conserved with P-Rex1.***A*, domain layout of P-Rex1 in gray and P-Rex2 colored by domain. The sequence identities between domains of the isoforms are labeled. *B*, schematic of the domain organization of P-Rex1. *C*, Cryo-EM reconstruction of P-Rex2 (composite map) colored by domain with atomic model superimposed. The IP4P subdomain was not resolved in P-Rex2, unlike in P-Rex1.

Both enzymes are maintained in a cytosolic, autoinhibited state with limited basal activity until they are recruited to the cell membrane and activated by the lipid PIP_3_ and the Gβγ subunits of heterotrimeric G proteins (9, 13, 23). Structural studies of P-Rex1 have begun to clarify how accessory domains facilitate autoinhibition and how Gβγ and PIP_3_ relieve this (5, 24, 25, 26, 27, 28, 29). A two-pronged autoinhibitory mechanism is mediated by interdomain contacts that restrict the DH domain from interacting with GTPases (24, 25). One interaction is formed by the DEP1 domain docking against the DH domain through a hydrophobic pocket of residues to stabilize a compact, low activity state (27) (Fig. 1*B*). A second interaction is mediated through the PH domain, engaging the 4HB of the IP4P (24, 25). PIP_3_ binding to the PH domain disrupts the PH–IP4P contact—an effect that the soluble headgroup of PIP_3_, IP_4_, does not reproduce (25). In fact, IP_4_ stabilizes the PH–IP4P autoinhibitory conformation. Gβγ, which binds to the surface formed by the DEP2, PDZ1, PDZ2 and IP4P domains, likely contributes to activation by promoting membrane localization, but this has not been conclusively determined (26). Together, PIP_3_ and Gβγ bind to synergistically activate P-Rex1 and fully relieve autoinhibition. Despite the wealth of structural information available on P-Rex1 (5, 24, 25, 26, 27, 30), there is only a single reported structure of a piece of P-Rex2: the isolated PH domain (31).

While the close sequence similarity and shared domain architecture suggest that P-Rex2 adopts a structure similar to P-Rex1, there are notable differences between the two in substrate specificity and regulation (8, 32). For example, P-Rex1 can activate all Rac-like and some Cdc42-like GTPases *in vitro*, but only Rac-like GTPase activation has been observed *in vivo* (6, 9, 32, 33). On the other hand, P-Rex2 specificity *in vitro* seems to be limited to Rac1 (23, 34). Additionally, the tumor suppressor protein phosphatase and tensin homolog (PTEN) has been shown to form a co-inhibitory interaction with P-Rex2, but not P-Rex1 (10, 35, 36). Furthermore, P-Rex1 contains a membrane localization element in the β3/β4 loop of its PH domain that is missing in P-Rex2. When this element is swapped into the corresponding loop of P-Rex2, it enhances membrane localization of P-Rex2 (5). Collectively, this evidence suggests that there may be differences in the molecular features of P-Rex1 and P-Rex2 that lead to these unique properties. In support of this, cross-linking mass spectrometry data on P-Rex2 (36) do not entirely correlate with the autoinhibited structure of P-Rex1, as P-Rex1 homologs of some cross-linked P-Rex2 residues are too far apart—much beyond the length of the crosslinker utilized (Fig. S1*A*). These data point to potential structural differences between the two proteins that have not yet been described.

Here, we investigated the structure and autoinhibitory mechanisms of P-Rex2 using an integrative structural biology approach. We determined a moderate-resolution cryo-electron microscopy (cryo-EM) structure of full-length P-Rex2 which showed that, although the N-terminal module and C-terminal core each individually exhibit a similar domain organization as compared to P-Rex1, the N-terminal module itself is positioned proximally to the core, with the DEP1 domain contacting the base of the IP4P domain. Furthermore, hydrogen-deuterium exchange mass spectrometry (HDX-MS) experiments show that, unlike P-Rex1, binding of IP_4_ has no effect on the overall conformation of P-Rex2. Size-exclusion chromatography coupled with small-angle X-ray scattering (SEC-SAXS) shows that although DEP1 promotes a compact overall architecture of the N-terminal module, it does not change the flexibility of the DH/PH tandem as previously described in P-Rex1. Despite these differences, mutagenesis and biochemical assays demonstrate that the DEP1 domain remains essential for autoinhibition by engaging the DH domain similarly to P-Rex1. This work reveals unique structural features that distinguish P-Rex2 from P-Rex1, providing insights into key molecular details that may confer regulatory differences between the two.

## Results

### The overall structure of P-Rex2 resembles that of P-Rex1, with differences in the positioning of the N-terminal module

To begin to understand the molecular differences in P-Rex2 as compared to P-Rex1, we determined the structure of full-length (FL) P-Rex2 using cryo-electron microscopy (cryo-EM) single particle analysis (Figs. 1*C*, S2–S5, & Table 1). Similar to P-Rex1, P-Rex2 exhibits a strong preferred orientation on grids (Figs. S2*C* and S4), necessitating the collection of supplemental data at a 35° tilt to provide additional views of the particle. Still, this was not enough to completely overcome the preferred orientation problem, and this coupled with inherent flexibility within P-Rex2 led to anisotropy within our maps. The overall domain organization of P-Rex2 resembles that of P-Rex1, composed of an N-terminal catalytic module (DH/PH-DEP1) and a C-terminal regulatory core (DEP2-IP4P) (Fig. 1*C*). Early-stage data processing led to a reconstruction of the whole particle with a global resolution of 3.4 Å according to cryoSPARC. This map was used to dock in starting models generated by AlphaFold (Figs. S3*A* and S4*A*). However, resolution of the N-terminal module was very low in comparison to the C-terminal core of the protein, in part due to the flexibility of the N-terminus relative to the core. In subsequent data processing pipelines, we focused on generating maps of the C-terminal core and the N-terminal module independent of each other, in attempts to achieve more interpretable maps. This led to two final maps: one in which the core was resolved to higher resolution overall, exhibiting a global resolution of 2.9 Å according to cryoSPARC (Figs. S3*B* and S4*B*), and one where the N-terminal module was more interpretable with a global resolution of 4.4 Å (Fig. S4*C*). However, these resolutions are overestimated, as the gold standard FSC curves have gradual slopes with rebounding peaks around 5 Å due to both the flexibility of P-Rex2 and its orientation bias (Fig. S4). Based on inspection of the maps, estimates from 3DFSC (37) are likely more accurate with the resolution of the core and N-terminal module at 3.6 Å and 6.8 Å, respectively (Fig. S4, *B* and *C*).

**Table 1:** Cryo-EM data collection, processing, and modeling Statistics

Data collection & Pre-processing	Untilted	Tilted
EMPIAR accession code	EMPIAR-13635	
Micrographs (collected)	12,070	7722
Electron dose (e^-^/Å^2^)	50	50
Frames per movie	1143	1206
Voltage (kV)	300	300
Stage tilt (°)	0	35
Pixel size (Å)	0.7296	0.7296
Defocus range (μm)	0.7–2.2	0.7–2.2
Micrographs (used)	8133	6959
Extracted particles	2,184,088	2,137,912
Final particles after 2D classification	1,390,781	1,585,976


Data processing	Composite map	Consensus map	N-terminal local refinement	Whole particle local refinement
EMDB accession code	EMD-74550	EMD-74547	EMD-74548	EMD-74549
Symmetry imposed	C1	C1	C1	C1
Particles in final map	-	215,042	101,178	128,298
Global Resolution (Å, at 0.143)	-	2.9	4.4	3.4
3DFSC sphericity	-	0.734	0.872	0.83
Map sharpening B factor (Å^2^)	-	79.8	161.7	76.3


Atomic modeling	
PDB accession code	9ZQ7
Model Composition	
Chains	1
Non-hydrogen atoms	8320
Hydrogens	7704
Protein residues	1035
Water	0
Ligands	0
*B* factors (Å^2^, min/max/mean)	
Protein	30/524/132
Ligand	0
Water	0
RMS deviations	
Bond lengths (Å)	0.003
Bond angles (°)	0.477
Validation	
MolProbity score	1.63
Clashscore	6.97
Rotamer outlliers (%)	1.53
CaBLAM outliers (%)	0.86
Ramachandran plot (%)	
Favored	97.5
Allowed	2.4
Outliers	0.1
Model vs. Data^a^	
CC mask	0.58
CC box	0.7
CC peaks	0.44
CC volume	0.6
Map-to-model FSC (Å, at 0.5)^a^	3.4
EMRinger score^a^	1.87
Q-score^a^	0.37

a Composite map.

**Figure 2:**
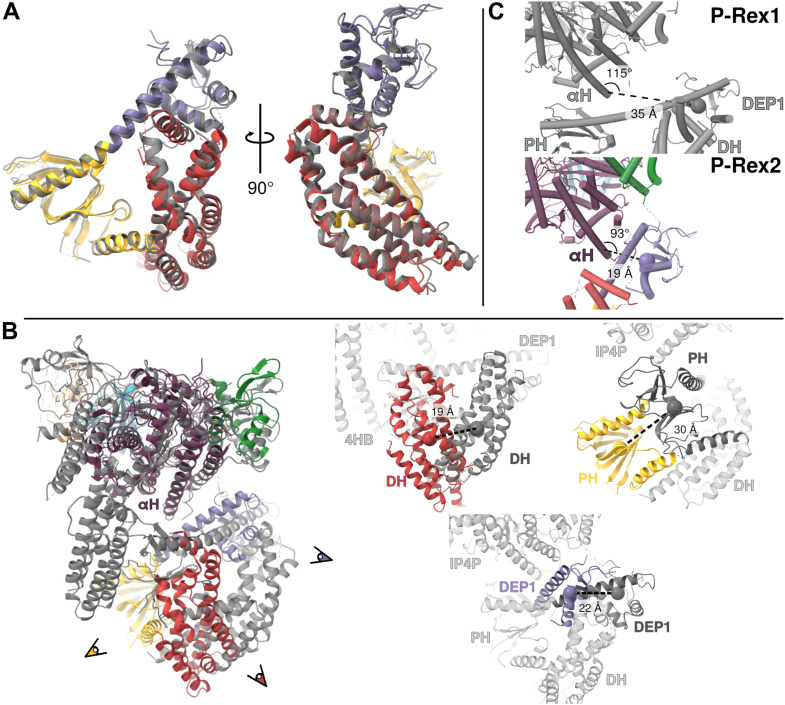
**P-Rex2 shows a relative shift in the position of the N-terminal module as compared to P-Rex1.***A*, N-terminal modules of P-Rex1 (*gray*, PDB: 8TUA, residues 38–499) and P-Rex2 (colored according to domain, residues 15–467) aligned to one another. There are minimal differences in their overall architecture. *B*, P-Rex2 aligned to P-Rex1 through the αH helix in the IP4P domain (P-Rex1 1491–1513, P-Rex2: 1431–1453) using Coot least-squares (LSQ) superimposition. A close-up view of each N-terminal domain (noted by the eyeballs) is shown in the insets, with P-Rex1 shown in gray. Spherical markers denote the center of mass (COM) of each homologous domain, and the distance between these in the aligned structures is indicated (PRex1 DH 38–237, PH 238–396, DEP1 397–499; P-Rex2 DH 15–211, PH 212–365, DEP1 366–467). *C*, the distance between the COM of the DEP1 domain and L1513/L1453 of the αH helix for P-Rex1 and P-Rex2, respectively, is shown. The measurement for an angle formed by a line connecting these two points and a line connecting L1513/L1453 to Q1491/A1431 of the αH helix for P-Rex1 and P-Rex2, respectively, is shown. Together, these describe the degree to which the N-terminal module is rotated and translated closer to the core in P-Rex2 as compared to P-Rex1.

Like in autoinhibited P-Rex1, the P-Rex2 DH domain is connected to the PH domain through a bent helix, while the PH domain is connected to the DEP1 domain through a more linear, continuous helix, and the DEP1 domains docks onto the end of the DH helical bundle (Figs. 1*C*, 2 and S5, *B*–*D*). The C-terminal core of P-Rex2 is composed of the second DEP domain, both PDZ domains and the IP4P domain where the IP4P domain is the central component and is decorated with the other accessory domains at peripheral ends. A β-strand connects DEP2 to PDZ1 and is part of a large β-sheet that twists through the center of the IP4P domain from one side to the opposite. The domain organization within each the N-terminal module and C-terminal core is conserved between P-Rex1 and P-Rex2, exhibiting an RMSD Cα of 1.4 Å each (Fig. 2). However, the P-Rex2 IP4P subdomain, observed in the autoinhibited P-Rex1 structures, is not resolved in any of our 2D class averages or 3D reconstructions.

Despite the overall structural similarity in P-Rex1 and P-Rex2, there is a significant shift in the N-terminal module relative to the core such that the P-Rex2 DEP1 domain is positioned proximal to the αH helix at the base of the core rather than distally to the side (Fig. 2, *B* and *C*). To quantify this relative shift, the αH helices were aligned (RMSD Cα of 0.32 Å), and the centers of mass (COMs) of each N-terminal domain were used to measure the distances between homologous domain pairs in the N-terminus. Comparing P-Rex1 and P-Rex2, the DH, PH, and DEP1 domains are shifted by 19 Å, 30 Å, and 22 Å, respectively (Fig. 2*B*). The P-Rex2 DEP1 domain COM is ∼16 Å closer to the base of the αH helix than the P-Rex1 DEP1 COM (Fig. 2*C*). Additionally, the angle of the DEP1 COM from the αH helix decreases from 115° in P-Rex1 to 93° in P-Rex2, resulting in an overall more compact structure of P-Rex2 (Fig. 2*C*). Given this substantial relative shift in the N-terminal module, we mapped the existing P-Rex2 cross-linking mass spectrometry data (36) onto the P-Rex2 structure to assess how well the data corroborated the N-terminal module position (Fig. S1). Distances between residues in the N-terminal module cross-linked to residues in the core are mostly within reason, based on the length of the cross-linker used, especially in comparison to the distances between homologous residues in P-Rex1 (Fig. S1). However, there are some exceptions. For example, K337 and K364 in the PH domain are too far away from K1442 in the IP4P domain. These cross-links also occur at low frequency in comparison to the others, which could suggest that these represent non-specific intermolecular interactions between different P-Rex2 molecules. Alternatively, given its flexible nature, these cross-links may represent transient or low-population conformations of P-Rex2 that are not represented in our final cryo-EM maps.

### Structural differences in the proposed phosphatase active site and Gβγ-binding elements

When the structure of the P-Rex1 C-terminal core was first determined in complex with Gβγ, it was discovered that the IP4P domain is structurally very similar to *Legionella* bacterial phosphoinositide 3-phosphatases (26). Based on homology, the catalytic triad in P-Rex1 was proposed to be formed by Cys1583, Arg1589, and Asp1638 (Fig. S5*F*), although, to date, no substrates have been identified to confirm P-Rex1 phosphatase activity. P-Rex2 also shares this phosphatase fold; however, there are notable differences in the homologous residues that would form the catalytic triad. Although Cys1529 and Arg1535 are structurally conserved, Asp1584 is pointed away from the other triad residues, albeit map density in this area is weaker than in the rest of the “active site.” Intriguingly, Cys1529 seems to form a disulfide bond with Cys1536 although the P-Rex2 cryo-EM sample was kept under reducing conditions at all times (Fig. S5, *E* and *G*). The corresponding residue in P-Rex1 is Ser1590, which would not be able to form a disulfide bond (Fig. S5*F*). Although phosphatase activity has not yet been evaluated for P-Rex2, these differences would likely preclude any activity at this site.

On P-Rex1, Gβγ binds the “top” of the C-terminal core, burying residues on the IP4P domain and both PDZ domains. Preceding the loop containing Asp1638 is a helix that forms part of this site and is important for engaging Gβγ (26) (Fig. S5*F*). In P-Rex2, the C-terminus of this helix and the beginning of the loop on which Asp1584 resides are disordered, and the remainder of the loop is structurally different from that of P-Rex1 (Fig. S5*G*). Additionally, parts of P-Rex1 PDZ1 and PDZ2 that are buried by Gβγ are disordered in P-Rex2. Because these structural elements collectively are components of the Gβγ-binding site, these divergences could have implications in P-Rex2 interacting differently with Gβγ as compared to P-Rex1. However, Gβγ binding to P-Rex2 has not yet been structurally characterized or quantitatively measured to determine if these structural differences lead to differences in binding affinity.

### IP_4_ binding does not enable resolution of the P-Rex2 IP4P subdomain

In P-Rex1, the IP4P 4HB subdomain extends from the C-terminal core and makes contact with the N-terminal module, promoting autoinhibition (24, 25). This closed, autoinhibited conformation is stabilized by binding of inositol-(1,3,4,5)-tetrakisphosphate (IP_4_), the soluble head group of the lipid PIP_3_, to the PH domain (25). Addition of IP_4_ to the P-Rex1 cryo-EM sample facilitated resolution of both the 4HB and the N-terminal module by stabilizing the contact between the two, as these elements appear to be otherwise flexibly tethered to the core (26). While the N-terminal module was clearly visualized in our P-Rex2 cryo-EM data, the IP4P subdomain was never resolved in any 2D class averages or 3D reconstructions despite extensive particle classification aimed at isolating subsets of particles wherein the subdomain might be observed (Figs. S2*C* and S3). The subdomain (∼29 kD) was confirmed to be intact in the P-Rex2 protein sample *via* molecular weight estimation by SDS-PAGE (Fig. S2*A*). Seeking to resolve the P-Rex2 IP4P subdomain, we added a 4-fold molecular excess of IP_4_ to the P-Rex2 cryo-EM sample and collected a small dataset. However, this did not alter the resulting 2D class averages or 3D reconstructions, and we were still unable to resolve the subdomain (Fig. S6). This suggests that IP_4_ binding does not affect the conformation of P-Rex2 as it does P-Rex1.

### Binding of IP_4_ does not affect the tertiary structure of P-Rex2

**Figure 3:**
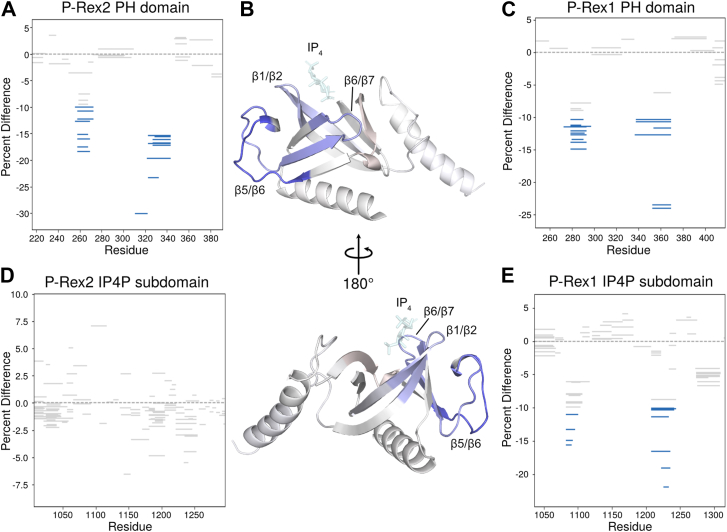
**IP_4_ does not alter the dynamics of the P-Rex2 IP4P subdomain, unlike P-Rex1.** HDX-MS data were collected on P-Rex2 in the presence and absence of IP_4_. *A*, HDX-MS peptide data at the 10,000 s time point shown on a Wood’s plot. Changes in protection greater than or equal to 10% are colored in blue. These changes indicate less deuterium uptake in the presence of IP_4_. The PH domain of P-Rex2 (216–381) exhibits IP_4_-dependent protection in the PIP_3_-binding site. *B*, P-Rex2 PH model colored according to difference HDX-MS data at the 10,000 s time point. Blue and light red regions indicate less and more deuterium uptake, respectively, in the presence of IP_4_. IP_4_ is docked based on the P-Rex1 PH•IP_4_ structure (PDB: 5D3X) and shown transparently in cyan. Loops in between β-strands are labeled. *C*, HDX-MS data from Ravala *et al.*, 2024 shown on a Wood’s plot for comparison. P-Rex1 PH (247–418) shows protection from deuterium uptake in the PIP_3_-binding site in the presence of IP_4_. *D*-*E* the IP4P subdomain of P-Rex1 (1036–1316) shows increased protection from deuteration in the 4HB_1_ and 4HB_2_ helices. However, the P-Rex2 IP4P subdomain (996–1296) does not show any significant change in deuteration uptake in the presence of IP_4_.

To further examine the lack of effect of IP_4_ on visualization of the IP4P subdomain, we conducted hydrogen-deuterium exchange mass spectrometry (HDX-MS) on P-Rex2 in the presence and absence of IP_4_. HDX-MS measures deuterium uptake into a sample of protein on a per-peptide basis, allowing assessment of the structure and dynamics of the protein. By comparing samples of P-Rex2 with and without IP_4_, we can assess how its structure changes upon IP_4_ binding. Like in our cryo-EM experiments, we used a 4-fold molar excess of IP_4_ relative to P-Rex2. In P-Rex2, IP_4_ binding protects the β1/β2 and β6/β7 loops as well as the β6 strand from deuterium uptake through direct binding to the PIP_3_-binding site (Fig. 3, *A* and *B*). Similarly, IP_4_ binding to P-Rex1 strongly decreases deuterium uptake in these structural elements (Fig. 3*C*; reanalyzed data from Ravala *et al.*, 2024). However, it also decreases exchange in the P-Rex1 PH β5/β6 loop and IP4P 4HB_1_ and 4HB_2_ helices by stabilizing the interaction between the PH domain and the 4HB (25) (Fig. 3, *C* and *E*). Interestingly, the P-Rex2 PH β5/β6 loop also exhibits a strong decrease in deuterium uptake, yet P-Rex2 lacks any significant IP_4_-dependent protection within the IP4P subdomain or any other regions of the protein (Fig. 3, *A*, *B* and *D*). This suggests either that IP_4_ does not affect contacts between the N-terminal module and IP4P subdomain or that these interactions do not occur. In comparing the HDX-MS profiles of the IP4P subdomain of P-Rex2 and the P-Rex1 4HB in the absence of IP_4_, the deuterium incorporation dynamics are very similar (Fig. S11) (25). This implies that these two regions may be structurally related to one another, although this has yet to be definitively determined.

### P-Rex1 is more active than P-Rex2 against Rac1

**Figure 4:**
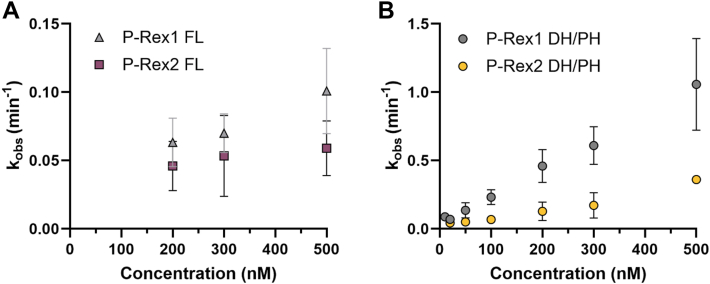
**P-Rex2 exhibits lower basal activity against soluble Rac1 than P-Rex1.***A*, *in vitro* GEF activity assay with purified full-length P-Rex1 and P-Rex2. *B*, GEF activity of purified P-Rex1 and P-Rex2 DH/PH constructs assessed at different concentrations. Error bars indicate standard deviation.

The distinct structural organization of P-Rex2 prompted us to investigate how its basal GEF activity might be different from that of P-Rex1. Utilizing a guanine-nucleotide exchange assay that measures GEF activity through loading soluble Rac1 with a fluorescently-tagged, nonhydrolyzable mant-GTP, we compared the activities of P-Rex2 and P-Rex1 constructs. Both P-Rex1 and P-Rex2 exhibit concentration-dependent activity levels where, at higher concentrations, P-Rex1 activity on Rac1 exceeds that of P-Rex2. At 500 nM GEF, we observe a ∼1.7-fold difference in activity between FL P-Rex1 and P-Rex2 (Fig. 4*A*). To tease out whether higher GEF activity arises from differences in specificity for Rac1 or autoinhibition from domains outside of the catalytic core, we purified the isolated P-Rex DH/PH tandems and analyzed the activity of each (Figs. 4*B* and S7*A*). Again, P-Rex2 DH/PH displayed notably lower activity than the P-Rex1 DH/PH fragment, with the greatest difference in calculated k_obs_ at a single concentration being ∼3-fold, showing that there is a difference in intrinsic activity of each for Rac1. Compared to the FL P-Rex constructs, the isolated DH/PH tandems of P-Rex1 and P-Rex2 showed ∼10- and 6-fold increases in activity, respectively, highlighting that the domains outside of DH/PH do exert autoinhibition against the catalytic tandem. Together, these observations indicate that both specificity for Rac1 and the autoinhibitory controls imposed by the accessory domains in the full-length protein likely play a role in measured differences in P-Rex1 and P-Rex2 activities. Because the fold differences in activities of the DH/PH and FL proteins are ∼3 *versus* only ∼1.7, respectively, it appears that P-Rex1 might be more tightly regulated by autoinhibition. To pick apart these two effects more thoroughly, we attempted to compare the activities of these constructs against Rac2. However, although FL P-Rex1 showed about the same activity against Rac2 as Rac1, FL P-Rex2 showed almost no basal activity against Rac2 in our assay (data not shown). Therefore, although there are likely differences in basal activity and autoinhibition in P-Rex proteins, with P-Rex1 possibly being more tightly regulated, divergences in GTPase specificity between the two make these differences difficult to interpret.

### The P-Rex2 DH/PH takes on a single, compact conformational state independent of DEP1

**Figure 5:**
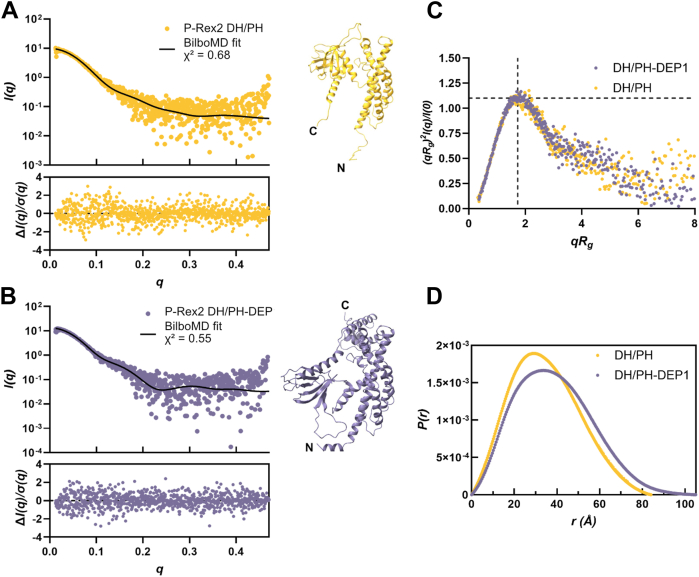
**SEC-SAXS data show that P-Rex2 DH/PH takes on a single, compact state.***A-B*, scattering intensity plots of P-Rex2 DH/PH (*yellow*) and DH/PH-DEP1 (*purple*) fit with the BilboMD model. The output BilboMD model is shown to the *right* of the plot. Normalized fit residuals are shown in the *bottom panel*. *C*, dimensionless Kratky plot for P-Rex2 DH/PH and DH/PH-DEP1. There is no significant change in shape between the DH/PH and DH/PH-DEP1. *D*, Pair distance distribution function of P-Rex2 DH/PH and DH/PH-DEP1.

Previous size-exclusion chromatography coupled with small-angle X-ray scattering (SEC-SAXS) experiments showed that the P-Rex1 DH/PH exists in two conformational states—the extended and compact form—whereas the DH/PH-DEP1 construct favors a single, compact conformation (27). To determine if the P-Rex2 DEP1 domain also induces compaction of the catalytic core, we performed SEC-SAXS analysis on purified P-Rex2 DH/PH and DH/PH-DEP1 constructs (Fig. S7*A*). The SAXS profiles of both P-Rex2 constructs were consistent with monodisperse, well-folded particles (Fig. 5, *A* and *B*, Table 2). Dimensionless Kratky analysis confirmed that both P-Rex2 DH/PH and DH/PH-DEP1 are compact and folded, exhibiting nearly identical profiles with well-defined peaks characteristic of globular proteins (Fig. 5*C*). Guinier analysis yielded correlation volumes corresponding to molecular weights of 41.6 and 57.1 kDa, respectively, within reasonable agreement with the expected molecular weights (44 and 51 kDa) (Fig. S7, *B* and *C*). The pair distance distribution functions, P(r), indicated D_max_ values of 84 Å for P-Rex2 DH/PH and 105 Å for P-Rex2 DH/PH-DEP1 which suggests that the maximum particle dimension increases between the two constructs reasonably with the increase in mass (Fig. 5*D*). This is not the case in P-Rex1 where the maximum particle dimension of P-Rex1 DH/PH, ∼105 Å, is roughly the same as the DH/PH-DEP1, 104 Å^27^. Together, this suggests that the P-Rex2 DH/PH may not take on an extended state like the P-Rex1 DH/PH and that it favors compact states regardless of the presence of DEP1. To directly compare the data from P-Rex1 and P-Rex2, we analyzed the published P-Rex1 SAXS datasets (SASDHY9 and SASDHW9) and our P-Rex2 datasets using BilboMD (38) rather than the previously employed ensemble optimization method (EOM) (39). Using the AlphaFold server to generate an initial model of each construct and BilboMD to produce ensemble models that represent highly populated conformations in solution, we identified that both P-Rex2 constructs were best fit by a single-state model (Fig. 5, *A* and *B*). However, when we performed the BilboMD analysis on the P-Rex1 data (Fig. S8), BilboMD did not reproduce clear indication of two discrete conformations of the P-Rex1 DH/PH, as previously shown with EOM analysis (27), and only suggested a more elongated state corresponding to a larger apparent molecular weight than would be expected for DH/PH. In all, these data suggest that both P-Rex2 DH/PH and DH/PH-DEP1 exist predominantly in compact, folded conformations in solution, indicating potentially reduced conformational flexibility as compared to P-Rex1.

**Table 2:** P-Rex2 SEC-SAXS data analyses

Data metric	P-Rex2 DH/PH (SASDZV5)	P-Rex2 DH/PH-DEP1 (SASDZU5)
Guinier analysis		
Rg (Å)	26.12 ± 0.38	29.11 ± 0.47
I(0)	9.68 ± 0.11	13.27 ± 0.17
Q_min_ ∗ Rg	0.455	0.684
Q_max_ ∗ Rg	1.312	1.301
Molecular weight analysis		
Porod Volume (Å^3^)	52,300	77,200
MW (Vc) (kDa)	41.6	57.1
Shape (S&S)	Compact	Compact
D_max_ (Å)	81.1	96.0
P(r) analysis		
D_max_ (Å)	84	105.0
Rg (Å)	26.94 ± 0.16	29.99 ± 0.28

### The P-Rex2 DEP1 domain plays a role in maintaining autoinhibition

**Figure 6:**
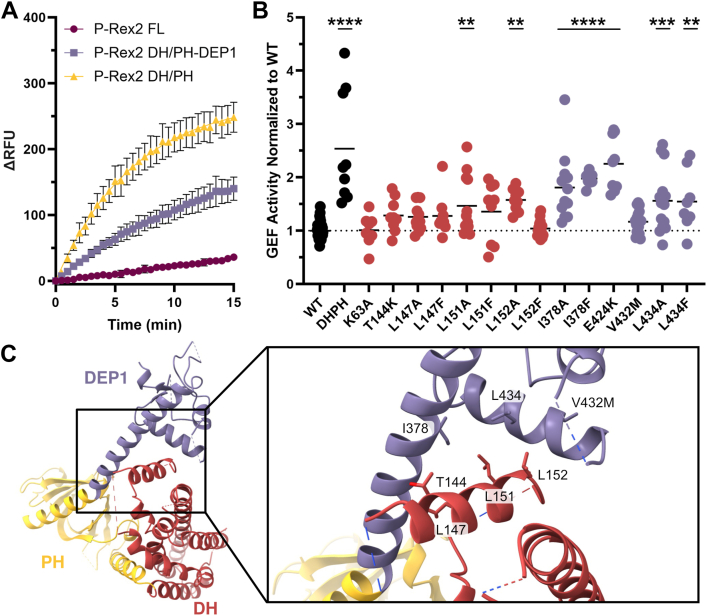
**The P-Rex2 DEP1 domain exerts autoinhibition on the catalytic core *via* interactions with the DH domain.***A,in vitro* GEF activity assay with soluble Rac1 plus P-Rex2 FL (300 nM), DH/PH-DEP1 (300 nM), and DH/PH (200 nM). Error bars represent standard deviation. *B,* GEF activity assay with soluble Rac1 plus P-Rex2 DH/PH-DEP1 variants, colored according to the domain in which the mutation lies. *C*, residues that were evaluated for their importance in maintaining autoinhibition through the DH–DEP1 interface.

Previously, the DEP1 domain was shown to play an important role in maintaining P-Rex1 autoinhibition through contacts with the DH domain (24, 25). While P-Rex2 is structurally highly conserved within the N-terminal module, SEC-SAXS data alluded to potential differences in flexibility and conformation within the isolated DH/PH domain. This led us to investigate the role of DEP1 in P-Rex2 autoinhibition. We first compared the activities of P-Rex2 DH/PH, DH/PH-DEP1, and FL constructs using the guanine-nucleotide exchange assay with soluble Rac1 (Fig. 6*A*). As expected, P-Rex2 FL exhibited the lowest activity. The DH/PH-DEP1 construct exhibited intermediate activity between the full-length and DH/PH constructs, consistent with the DEP1 domain being involved in autoinhibition. Next, we introduced single-point mutations in the DH/PH-DEP1 construct at the DH–DEP1 interface. Because the resolution of the N-terminal module in the EM structure was not high enough to resolve sidechains, we focused on homologous residues that are important for maintaining P-Rex1 autoinhibition (25) and compared the activities of these variants to those of wild-type DH/PH-DEP1 and DH/PH (Fig. 6, *B*, *C* and S9). Variants L151A, L152A, L434A, and L434F exhibited moderately increased activity, whereas I378A, I378F, and E424K showed the highest increases in activity. These data support that the hydrophobic pocket formed by Leu151, Leu152, Ile378, and Leu434 as well as a potential ionic contact at Glu424 collectively contribute to stabilization of the interface between the DH and DEP1 domains to promote autoinhibition (Fig. 6*C*). The K60A and K63A variants were generated due to the proximity of these residues to Glu424 in our predicted model before we obtained the cryo-EM structure of P-Rex2; however, we were unable to build these in our experimental model. The K60A variant did not express, and the L434 variants consistently exhibited low expression, which may reflect their importance in the stability of the N-terminal module (Fig. S9).

## Discussion

**Figure 7:**
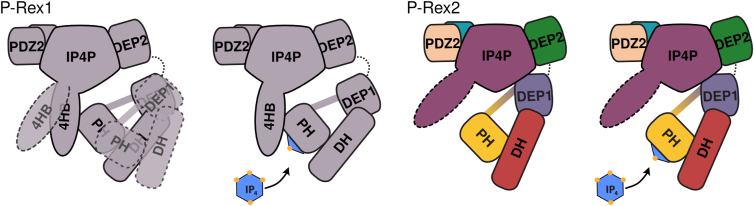
**Overview of structural differences between P-Rex2 and P-Rex1.** Although the structure of each the N-terminal module and C-terminal core are conserved between P-Rex2 and P-Rex1, the positioning of the two relative to one another is divergent. Furthermore, the autoinhibited form of P-Rex1 exhibits a conformation where the N-terminal module interacts with the 4HB subdomain of the IP4P domain, both of which appear to be otherwise flexibly tethered to the C-terminal core. This conformation is stabilized by binding IP_4_. In contrast, the P-Rex2 N-terminal module docks to the bottom of the C-terminal core and lacks any interaction with the IP4P subdomain which was not resolved in our cryo-EM data, presumably because it is flexibly tethered to the core. IP_4_ binding has no apparent effect on the structure or dynamics of P-Rex2 outside of the PIP_3_-binding site.

Here, we presented the first structural characterization of autoinhibited P-Rex2 and revealed how its domain organization and regulatory features diverge from its close family member, P-Rex1 (Fig. 7). Our cryo-EM structure shows that while both the N- and C-termini are each individually structurally well-conserved between P-Rex1 and P-Rex2, the positions of these relative to one another are strikingly different between the two proteins. This major architectural dissimilarity may have significant consequences for how autoinhibition is achieved, pointing to potentially unique mechanisms being utilized in each isoform.

In P-Rex2, both the helix that spans the PH to DEP1 domains and the DEP1 domain itself are proximal to the base of the IP4P core. Docking the N-terminal module to the core likely enables its resolution in our cryo-EM structure by tethering the otherwise likely flexible N-terminus. However, it is difficult to pinpoint the underlying cause of this structural difference between P-Rex proteins. The surface of the P-Rex2 N-terminal module that interacts with the core is very similar to that of P-Rex1, suggesting that this is not the source of the divergence. One hypothesis is that the connection between the first and second DEP domains in P-Rex2 is more rigid and inflexible than that in P-Rex1, as this linker is composed of bulkier, less flexible residues in P-Rex2 (TFYPRNE) compared to P-Rex1 (TYKARSE). This is supported by clear map density representing the P-Rex2 DEP1-DEP2 connection, although it is not high enough resolution for us to be able to confidently model into it. Alternatively, the unique structural features at the base of the P-Rex2 core could promote the docking of the N-terminal module. In P-Rex1, the IP4P αH and αI helices are moderately well-defined and are connected by a disordered loop of ∼14 residues (Fig. S10*A*). In P-Rex2, the homologous residues are much more well-resolved, forming a helical structure that packs very closely to the αH helix (Fig. S10, *B* and *C*). Although the functional consequences of these structural features remain unclear, elucidating whether these elements contribute to autoinhibition could provide insight into regulatory surfaces that could potentially be exploited for P-Rex-specific inhibition.

Contrary to the P-Rex1 autoinhibitory mechanism, P-Rex2 does not exhibit a PH–IP4P subdomain interaction, with or without IP_4_. IP_4_ is an abundant negative regulator of neutrophil activation (40) as it can compete with PIP_3_ to bind to PH domains, thereby blocking signaling downstream of PIP_3_ (41). P-Rex1, which is strongly expressed in neutrophils (9), is likely inhibited in these cells through both this mechanism as well as IP_4_ stabilization of the P-Rex1 autoinhibited conformation. P-Rex2, however, is absent from neutrophils and other immune cells. Instead, P-Rex2 is expressed in Purkinje neurons (12) and liver, lung, kidney and pancreas cells (42), which utilize IP_4_ in a slightly different manner. In rat hepatocytes, for example, as little as 0.5 μM I(1,3,4,5)P_4_ extends the duration of I(1,4,5)P_3_-dependent Ca^2^^+^ signaling (43). While the concentration of IP_4_ in these cells is unknown, as it is quickly degraded in liver homogenate (44), given the sensitivity of the cells to small amounts of IP_4,_ the concentration of IP_4_ in hepatocytes is likely much less than that in neutrophils (4 μM) (45). This suggests that the differential regulation of P-Rex1 and P-Rex2 by IP_4_ may be an evolutionary biological adaptation to the specific cell types each protein is found in and the role IP_4_ plays in those cells.

While our HDX-MS data suggest that the P-Rex2 IP4P subdomain may take on a similar tertiary structure as P-Rex1, the position of the N-terminal module makes it unlikely that the IP4P subdomain would be able to make the same contacts with the PH domain, as the P-Rex2 PH domain is positioned farther away from the C-terminal core. Although our present data do not support a clear role for the P-Rex2 IP4P domain in autoinhibition, it may help contextualize data pointing to a role in PTEN-induced inhibition. Cross-linking mass spectrometry of P-Rex2 (36) shows limited intramolecular contacts between the IP4P subdomain and the N-terminal module. However, upon PTEN binding, there is a substantial increase in the number of cross-links between the IP4P subdomain and the DH and PH domains. This raises the possibility that PTEN does not primarily inhibit P-Rex2 through direct engagement with the N-terminus as previously suggested (10), but instead binds to the PDZ2 and IP4P subdomain (36) to strengthen long-range interdomain interactions between the IP4P and DH/PH. In this context, it is notable that the pancreatic cancer-associated V432M mutation, which lies near the DH–DEP1 interface, escapes PTEN-driven inhibition in breast cancer cells despite retaining PTEN binding (46). Our biochemical assays using the isolated N-terminal module show only a minimal, non-significant increase in basal nucleotide exchange activity in the V432 M variant, suggesting that this mutation does not drive disease through disruptions to autoinhibition from DEP1. However, these data imply that, in the full-length protein, the V432M mutation potentially changes how the N-terminal module interacts with the IP4P subdomain to disrupt PTEN-driven inhibition. While our understanding of the P-Rex2–PTEN coinhibitory complex is still limited, it would be valuable to investigate this mechanism further to identify how disruptions to this complex contribute to cancers and diabetes and, furthermore, to tease out why PTEN is a regulator of P-Rex2 and not P-Rex1.

We observed that the isolated P-Rex2 DH/PH exhibits slower nucleotide exchange on soluble Rac1 than that of P-Rex1. It is possible that this could be due to differences in specificity between the two GEFs for Rac1. Based on crystal structures of P-Rex1 DH/PH in complex with Rac1 or Cdc42, the α4/α5 loop and α5 helix of the DH domain may be involved in substrate specificity between different GTPases (5). P-Rex2 maintains very high sequence identity in these regions with a few exceptions. At the GTPase-binding site, P-Rex2 Val160 within the α4/α5 loop and Val167, Thr168, and Ile179 of the α5 helix are different from the homologous residues found in P-Rex1 (Ile186, Leu193, Ser194, and Leu205, respectively) and potentially could account for the observed differences in nucleotide exchange on Rac1. We also showed that, of the two identified mechanisms that govern P-Rex1 autoinhibition, only the DH–DEP1 interface is conserved in P-Rex2. However, the DH–DEP1 interface alone does not fully account for autoinhibition in P-Rex2, as P-Rex2 FL exhibits significantly lower activity than DH/PH-DEP1. This suggests that other regions of the protein must also participate in achieving full autoinhibition. We also observed differences in the Gβγ-binding sites between P-Rex1 and P-Rex2. However, because these regions are distal from both the P-Rex catalytic site and autoinhibitory regions, we do not believe these differences would influence P-Rex basal activity in the absence of Gβγ. Further examination of the C-terminal accessory domains for their possible roles in autoinhibition is needed. Additionally, biochemical and biological exploration of the alternative spliceoform P-Rex2b, which largely lacks the IP4P domain, has been very limited but might provide insight into a P-Rex2 that more closely exhibits the higher activity of P-Rex1.

Because of the unexpected differences in structure and autoinhibitory regulatory mechanisms, it is possible that P-Rex1 and P-Rex2 respond differently to activation by PIP_3_ and Gβγ. For example, interactions between the P-Rex1 PH domain and IP4P subdomain contribute to autoinhibition and must be disrupted for activation (25), whereas the lack of such coupling in P-Rex2 implies that the PH domain may be more readily available to bind to PIP_3_, allowing P-Rex2 to become activated at lower PIP_3_ concentrations despite exhibiting lower basal activity than P-Rex1. There are also potential differences in Gβγ binding. In P-Rex1, the region around Gln1615-Leu1647 was identified to play a major role in binding Gβγ (26). Within the homologous area in P-Rex2 (Lys1578-Pro1587), the structure diverges significantly. In another P-Rex1 element implicated in Gβγ binding (Met1582-Val1594), the homologous region in P-Rex2 appears to form an unusual disulfide bond that cannot form in P-Rex1 (Fig. S5, *F* and *G*). These differences suggest that P-Rex2 may bind Gβγ with a different mechanism and/or affinity than P-Rex1, potentially eliciting a distinct activation response. However, our understanding of the activation mechanisms of P-Rex RhoGEFs is still largely unexplored. Future work investigating this is still needed to understand how P-Rex1 and P-Rex2 escape their individual autoinhibitory mechanisms.

In summary, this work has begun to uncover how P-Rex2 regulation is unique from P-Rex1 and how its structural elements could contribute to these differences. Although both proteins share high sequence similarity, P-Rex1 and P-Rex2 autoinhibition are achieved in part through distinct domain organization. This highlights opportunities to selectively target one isoform over the other through divergent regulatory surfaces outside of the DH domain *via* the rational design of modulators. Attempts at specifically targeting individual Dbl RhoGEFs have historically been unsuccessful due to the flat, broad DH domain that is highly conserved and binds structurally similar GTPases. More broadly, these findings are exciting because they are proof of principle that even closely related Dbl RhoGEFs within the same subfamily can exhibit clear differences in their structure and regulation, offering a framework for designing selective modulators that target Dbl RhoGEFs by utilizing their accessory domains. Future efforts integrating structural and cellular studies will be essential for translating these mechanistic insights into strategies for therapeutically targeting misregulated RhoGEF signaling and, furthermore, fine-tuning GTPase signaling to treat and prevent disease.

## Experimental procedures

### Sequence identity comparison to P-Rex1

Using our P-Rex2 model, we identified the residue boundaries of each of the seven domains: DH: 15-211, PH: 212-365, DEP1: 366-467, DEP2: 473-569, PDZ1: 590-672, PDZ2: 673-815, and IP4P: 816-1606. The corresponding domain boundaries were identified in P-Rex1 based on structure comparison. Sequence identity was calculated using The Sequence Manipulation Suite (47).

### Cloning and plasmids

P-Rex1 full-length (FL), P-Rex1 DH/PH, and Rac1 DNA constructs were described previously (5, 26, 27). P-Rex2 pcDNA3.1/V5-His DNA was a gift from Ramon Parsons (Addgene plasmid #41555) (35). P-Rex2 FL was cloned into a pRK5 mammalian expression vector with a TEV-cleavable N-terminal GST tag and a C-terminal FLAG tag. P-Rex2 DH/PH (residues 1–377) and P-Rex2 DH/PH-DEP1 (residues 1–467) were each cloned into the pMALc2H_10_T vector with a TEV-cleavable N-terminal MBP-10xHis tag. P-Rex2 DH/PH-DEP1 point mutations were generated using site-directed mutagenesis.

### Protein production and purification

P-Rex1 and P-Rex2 FL were expressed in FreeStyle 293-F cells by transient transfection using polyethylenimine. Cells were harvested 48 h post-transfection by centrifugation for 15 min at 1000 × *g*, flash frozen in liquid nitrogen, and stored at −80 °C. Pellets were thawed and lysed in Cell Lytic M (Sigma) supplemented with 200 mM NaCl, 1 mM ethylenediaminetetraacetic acid (EDTA), 2 mM dithiothreitol (DTT), and a protease inhibitor cocktail (125 nM aprotinin, 6.8 μM leupeptin, 0.15 nM soybean trypsin inhibitor, 1 μM E-64, and 1 μM bestatin). After rocking at 4 °C for 15 min, lysates were clarified by ultracentrifugation at 40,000 rpm for 45 min at 4 °C in a Type 45 or 70 Ti rotor depending on the size of the sample. The supernatant was filtered through a glass fiber filter and incubated with 800 μl glutathione agarose resin per liter harvested cells for 1 to 2 h at 4 °C with gentle rocking. The resin was washed with 20 mM HEPES (pH 8), 200 mM NaCl, 1 mM EDTA, 2 mM DTT, and protease inhibitor cocktail. GST-tagged P-Rex proteins were eluted with 100 mM HEPES (pH 8), 300 mM NaCl, 1 mM EDTA, 1 mM DTT and 30 mM reduced glutathione. Overnight at 4 °C, elution fractions were digested with TEV protease (1:1 M ratio) and dialyzed against 20 mM HEPES (pH 8), 100 mM NaCl, 1 mM EDTA, and 2 mM DTT. Protein was then further purified over an ENrichQ (BioRad) anion exchange column in 20 mM HEPES (pH 8), 2 mM DTT, and eluted with a NaCl gradient from 100 mM to 550 mM over 30 CV. Purified protein was pooled, concentrated, and immediately used for experiments.

Soluble Rac1 was expressed in Rosetta (DE3) pLysS *E. coli* cells and purified as previously described (5). Truncated P-Rex constructs were also expressed in Rosetta (DE3) pLysS *E. coli* cells as N-terminal His-tagged MBP fusion proteins. Cells were grown in Terrific Broth to an OD_600_ of 0.8, induced with 0.1 mM IPTG at 16 °C, harvested after 16 to 20 h, and then flash frozen and stored at −80 °C. Cell pellets were thawed and resuspended in 9 ml 20 mM HEPES (pH 8), 300 mM NaCl, 0.1 mM EDTA, 2 mM DTT and protease inhibitor cocktail per 1 g cell pellet. Cells were then homogenized with a Dounce and lysed with an Avestin EmulsiFlex-C5 High Pressure Homogenizer. Lysate was clarified by ultracentrifugation at 40,000 rpm for 45 min in a Type 45 Ti rotor. The supernatants were then filtered through a glass fiber filter and incubated with Ni-NTA resin for 30 to 60 min. Resin was washed with buffer (20 mM HEPES, pH 8, 300 mM NaCl, 2 mM DTT) followed by buffer containing 10 mM imidazole, and proteins were eluted with buffer containing 250 mM imidazole. DH/PH and DH/PH-DEP1 elutions were then simultaneously dialyzed into buffer containing 20 mM HEPES (pH 7 or pH 8, respectively), 200 mM NaCl, 2 mM DTT, and 10% glycerol and digested with TEV protease overnight at a 1:2 M ratio of TEV: MBP-fusion protein to remove the N-terminal His-tagged MBP. Cleaved MBP-His was then captured by an additional pass over Ni-NTA resin. The flow-through was applied to a HiTrap SP Sepharose Fast Flow column (Cytiva) in 20 mM HEPES (pH 7) and 2 mM DTT and eluted over an NaCl gradient from 0 to 0.5 M over 4 CV.

P-Rex2 DH/PH-DEP1 mutants were expressed under the same conditions as the wild-type construct, but in small-scale 15 ml cultures. Cell pellets were resuspended in 1 ml 20 mM HEPES (pH 8), 300 mM NaCl, 0.1 mM EDTA, 2 mM DTT, and protease inhibitor cocktail. Resuspended cells were sonicated on ice (5 s on, 5 s off, for 60 s per cycle for three cycles) using a Q125 sonicator (Qsonica Sonicators), and lysates were clarified by centrifugation at 21,300 × *g* for 45 min at 4 °C. The supernatants were incubated with Ni-NTA resin for 30 to 60 min at 4 °C. The resin was washed and proteins eluted, cut with TEV, and dialyzed the same way as the wild-type DH/PH-DEP1. Cleaved MBP-His was then captured by an additional pass over Ni-NTA resin. The resin was washed one additional time with a buffer containing 20 mM HEPES (pH 8), 300 mM NaCl, 2 mM DTT, and 10 mM imidazole. The combined flow through and wash were concentrated using a 10kD MWCO Amicon concentrator. Final protein concentrations were estimated by visualization on SDS-PAGE.

### Cryo-EM grid preparation and data acquisition

P-Rex2 samples at 0.4 mg/ml were briefly incubated with 0.07 mM n-Dodecyl-Beta-Maltoside (DDM) before applying a 4 μl sample to a glow-discharged 300-mesh Quantifoil (1.2/1.3) grid, blotted for 4 or 6 s with a blot force of 20, and plunge-frozen into ethane cooled with liquid nitrogen using a Vitrobot (Thermo Fisher Scientific) set to 4 °C and 100% humidity. Data were collected at the Pacific Northwest Center for Cryo-EM (PNCC) using EPU (Thermo Fisher Scientific) on a Krios transmission electron microscope operating at 300 keV with a Falcon 4i direct electron detector (Thermo Fisher Scientific) and SelectrisX. Datasets were collected on an untitled grid (12,070 micrographs) with beam image shift (BIS) and on a grid tilted by 35° (7722 micrographs) without BIS. Tilted data were collected to overcome a preferred orientation of the sample on the grids.

For the IP_4_ experiments, small datasets were collected on P-Rex2 samples at 0.7 mg/ml that were briefly incubated with 0.07 mM DDM in the presence or absence of 16.6 μM IP_4_ and frozen with the same freezing conditions described above. Approximately 1300 to 1400 micrographs were collected for each dataset using a Glacios transmission electron microscope operating at 200 keV with a K3 direct electron detector (Gatan, Inc.) and then processed up through 2D classification. Both samples yielded classes with similar views without distinguishable differences in P-Rex2 conformation, mass present, or resolution (Fig. S6).

### Cryo-EM data processing

Untilted and tilted datasets were preprocessed separately. Motion correction, contrast transfer function (CTF) estimation, and particle picking were performed in Warp (48). The default BoxNet2Mask model was retrained on P-Rex2 particles for particle picking in these datasets. Particle stacks were then imported into cryoSPARC (49) for further processing.

In our first processing pipeline (Fig. S3*A*), the untilted and tilted datasets were each separately put through cursory 2D classification to sort out obvious junk in each particle stack. Using only the best particles with clear secondary structure features from the tilted dataset, *ab initio* volumes were generated. The volume containing the whole particle (both N- and C-terminal components) was then used, in combination with decoy *ab initio* volumes, in separate heterogeneous refinements for both the tilted and untilted particle stacks. Particles that were sorted into the whole particle volume from both heterogeneous refinements were combined and underwent an additional round of heterogeneous refinement. A final round of 2D classification was used as a quality check where only the highest quality particles were used for non-uniform refinement (NU Refine). The resulting volume was used in a final heterogeneous refinement, deriving the final particle stack. The best volume and associated particles were then used in non-uniform refinement and subsequent local CTF refinement in cryoSPARC; however, the maps generated from these jobs had odd features where recognizable helices looked step-like. In an attempt to resolve this, we used 3D refinement with Blush regularization in Relion 5.0 (50), which was fairly successful in improving the appearance of these helices. Finally, the refined map and particles underwent local refinement in cryoSPARC with a mask around the whole particle. The resulting map was put through density modification using EMReady (51) to improve the interpretability of the map for use in model building (Fig. S5, *B* and *C*).

In the final processing pipeline (Fig. S3*B*), each particle stack was separately cleaned through multiple rounds of 2D classification to sort out obvious junk, and then particle stacks from the untitled and tilted data were combined. Because of strong orientation bias despite the addition of tilted data, there were a few class averages that would dominate the views observed in 2D classification. Therefore, in an iterative 2D classification approach, these well-resolved classes were separated for later use, obvious junk was discarded, and further 2D classification was done on the remaining particles, which helped to sort out more rare, good-quality particle views. After several rounds of this, we were left with a much larger cleaned particle stack than without using this approach. With these particles, *ab initio* reconstruction yielded one volume where both the C-terminal core and the N-terminal module were resolved and three volumes where mainly only the core was resolved. Only the volume with clear density for the N-terminal module was used for downstream processing. This volume was processed through non-uniform refinement (52). This reconstruction along with three decoy volumes created from “junk” particles were used in heterogeneous refinement against the cleaned particle stack, resulting in ∼100 k additional particles sorting into the “good” class. Additional non-uniform refinement of this stack and volume resulted in a 2.9 Å consensus map, which featured primarily only the core (Fig. S4*B*). Given the apparent continuous flexibility of the N-terminal module, 3D Variability Analysis and 3DFlex were used to attempt to better resolve this region, but neither of these programs improved results. Instead, we used a binary approach where focused 3D classification was performed on the consensus map and its particles using a mask around the N-terminal module to sort out particles where the N-terminus is in a single position with respect to the C-terminus. The class containing a visible N-terminal module from this job was then locally refined using the same mask around the N-terminal module which produced the N-term local refinement map. The consensus map and the map from local refinement of the N-terminal module were used to generate a composite map in Phenix, utilizing a 4.5 Å resolution cutoff (Fig. S5*D*).

### Model building, refinement, and validation

Primarily, two maps were used to build the P-Rex2 model, as each map was more interpretable in different areas. One of these was the composite map and the other was the density-modified map from EMReady, both of which are described above. Using the AlphaFold Server (53), P-Rex2 models of each the N-terminus (residues 1–472) and C-terminus (473–1606) were generated. Models were fit into the aligned maps using ChimeraX (54), and the best fitting model for each the N- and C-terminus was chosen for further model building and modification. Certain parts of the protein were rigid body fit into the map with little further modification, as resolution in these areas was low, including the DH, PH, and DEP1 domains. The density of the midpoint of the helix connecting the PH and DEP1 domains was sparse, and the EMReady map was particularly helpful in modeling this area (Fig. S5, *C* and *D*). The connection between the DEP1 and DEP2 domains was clearly visible in the map but not at high enough resolution to build unambiguously since it was very different from this region in P-Rex1 and in the AlphaFold models. The part of the map representing the C-terminal core was the highest resolution and most interpretable part of the map. However, the PDZ2 domain was particularly disordered, and much of this domain was left unmodeled. The IP4P subdomain observed in the autoinhibited structure of P-Rex1, termed the 4HB, was completely missing from the P-Rex2 map, presumably because it is flexibly tethered to the core. There were also a number of loops in the IP4P domain that were left unmodeled due to sparse or missing density. Importantly, conclusions regarding the model were only drawn from regions of the structure that were well-supported by map density. Parts of the model that were completely outside of map density were deleted in Coot 0.9.8.95 (55), and then each model was run through rigid body real-space refinement in Phenix 1.21.2-5419 (56) after adding hydrogens and with minimization_global enabled (57).

After this, the model was further modified and rebuilt in Coot using tight geometric restraints. Because map density was very sparse or low resolution in some regions, homology models P-Rex1–Gβγ (6PCV), P-Rex1•IP_4_ (8TUA), and P-Rex1 DH/PH–DEP1 (7RX9) were used to assist decision making during model building. In some areas, the AlphaFold P-Rex2 model was different from the homologous region in P-Rex1 but nicely fit the P-Rex2 map, while in others, the predicted and homology models clearly deviated from the path of the map. In regions where the map was ambiguous, the AlphaFold P-Rex2 model was left with limited modification when it fit the density reasonably well, corresponded to helical or β-strand regions, and was conserved in P-Rex1. In areas where the P-Rex2 map diverged from P-Rex1, such as in loops where the prediction was less reliable or the density path was not clear, the model was deleted. After the building was complete, regions of the structure that formed helices or β-sheets were manually confirmed and annotated, and Phenix real-space refinement was run using secondary structure restraints. Model validation was performed in Phenix (57) (Table 1 & Fig. S5*A*). EMRinger was also run within Phenix (58). The associated web interface was used to run the 3D FSC program to calculate sphericity and generate a 3D FSC histogram and directional FSC plot (37, 59). The Q score was calculated during the validation stage of data deposition (60). Structure figures were generated using ChimeraX (54) and PyMOL (https://www.pymol.org/). Some software was accessed through membership in the SBGrid Consortium (61).

### Hydrogen–deuterium exchange mass spectrometry

Prior to conducting hydrogen/deuterium exchange experiments, optimal quench conditions were determined to generate the best enzymatic peptide coverage map for P-Rex2 FL, as previously described (26, 62). Briefly, 3 μl purified P-Rex2 (2 mg/ml in 20 mM HEPES (pH 8), 150 mM NaCl, 2 mM DTT) was diluted with 9 μl of H_2_O Buffer at 0 °C, and then mixed with 18 μl of ice cold quench buffers containing 0.1 M Glycine (pH 2.4), 16.6% glycerol and varying concentration of GuHCl (0.08, 0.8, 1.6 and 3.2 M). The quenched samples were subjected to on-line proteolysis and LC/MS analysis. The best sequence coverage of P-Rex2 was obtained using the 1.6 M GuHCl quench buffer.

P-Rex2 exchange stock solutions (10.8 μM of P-Rex2 FL and 10.8 μM of P-Rex2 FL with 45 μM IP_4_) were prepared in 8.3 mM Tris, 150 mM NaCl, pH 7.2 and kept on ice. HDX experiments were initiated by adding 50 μl of exchange stock solutions (P-Rex2 FL or P-Rex2 FL-IP_4_) to 150 μl of D_2_O buffer (8.3 mM Tris, 150 mM NaCl, pDread7.2) and incubating for various time intervals (10, 100, 1000, 10,000 and 100,000 s) at 0 °C. At each time, 36 μl of exchange mixture was withdrawn and quenched with 54 μl of ice-cold quench buffer [0.1 M Glycine (pH 2.4), 1.6 M GuHCl]. Quenched samples were aliquoted and immediately frozen on dry ice. Non-deuterated and equilibrium deuterated control samples were also prepared for back exchange correction (63).

All frozen samples were later loaded onto a cryogenic autosampler and automatically thawed at 4 °C before digestion on an immobilized pepsin column (16 μl bed volume) at 25 μl/min. Proteolytic fragments were collected on a trap column and separated on an Acclaim PepMap RSLC C18 reverse phase analytical column (ThermoSci, 0.3 × 50 mm, 2 μm, 100 Å) using a linear acetonitrile gradient (5%–45% over 30 min). The effluent was directed into an OrbiTrap Elite Mass Spectrometer (ThermoFisher Scientific, San Jose, CA). Instruments settings were optimized to minimize the back-exchange (64). The data were acquired in either MS1 profile mode or data-dependent MS/MS mode. Peptide identification was done with the aid of Proteome Discoverer software (ThermoFisher). The centroids of the mass envelopes of deuterated peptides were calculated with HDExaminer (Sierra Analytics Inc) and then converted to corresponding deuterium incorporation with corrections for back-exchange (65).

### GTPase exchange activation assay

P-Rex GEF activity was analyzed through a FRET-based nucleotide exchange assay (66) measuring association of GTPase with fluorescently labeled N-methyl-anthraniloyl-GTP (mant-GTP; Abcam), a nonhydrolyzable GTP analog, in a black 384-well plate (Greiner). P-Rex construct plus 2 μM GTPase were added in 20 mM HEPES (pH 8), 100 mM NaCl, 5 mM MgCl_2_, 1 mM DTT, and 5% glycerol at room temperature. The reaction was initiated by adding 0.8 μM mant-GTP and immediately measured for fluorescence at Ex = 280 nm, Em = 450 nm at room temperature in 30 s intervals for 15 min on a SpectraMax M5 (Molecular Devices) plate reader. Fluorescence curves were then fitted to a one-phase association model using GraphPad Prism.

### Statistical analysis

All statistical analyses were performed in GraphPad Prism software on data from at least three independent experiments. All GEF activity assay data were fit to a one-phase association model. The k value obtained was used to represent k_obs_ where applicable. All P-Rex2 DH/PH-DEP1 variants were normalized to P-Rex2 DH/PH-DEP1 WT and then evaluated for statistical significance. Statistical significance was determined using a one-way ANOVA test with a *post hoc* Dunnett’s test for multiple comparisons. ∗*p* < 0.05, ∗∗*p* < 0.01, ∗∗∗∗*p* < 0.0001.

### Size-exclusion chromatography coupled with small-angle X-ray scattering

Small-angle X-ray scattering (SAXS) experiments were performed at the SIBYLS beamline 12.3.1 at the Advanced Light Source. This beamline has additional inline instrumentation and detectors coupled to a size-exclusion column (67, 68). Purified P-Rex2 DH/PH and DH/PH-DEP1 were concentrated to 2 mg/ml and then buffer exchanged into 20 mM HEPES (pH 7), 200 mM NaCl, 2 mM DTT, and 1% glycerol SEC running buffer. The X-ray wavelength was set to 1.127 Å and the sample-to-detector distance to 2100 mm, which gives scattering vectors (q) ranging from 0.01 Å^-1^ to 0.4 Å^-1^. The scattering vector is q = 4πsinθ/λ, where 2θ is the scattering angle. The SAXS flow cell was coupled to an Agilent 1260 Infinity HPLC system using a Shodex PROTEIN KW-802.5 SEC column equilibrated with the running buffer at a flow rate of 0.65 ml/min. For SAXS measurements, 2 sec X-ray exposures were collected continuously during a 25 min elution. All frames for analyses had one SAXS frame corresponding to the running buffer before the detection of a peak subtracted from each.

The radius of gyration (Rg) was calculated for each of the subtracted frames using the Guinier approximation: I(q) = I(0) exp(−q2Rg2/3) with the limits qRg < 1.3. The elution peak was compared to the integral of ratios to background and Rg relative to the recorded frame using the program RAW (69). Uniform Rg values across an elution peak represent a homogeneous sample. Final merged SAXS profiles, derived by integrating multiple frames at the elution peak, were used for further analyses. Calculated were the Guinier plot to provide information on the aggregation state, the volume of correlation (Vc) to estimate the molecular weight (70), and the pair distribution function [P(r)] to calculate the maximal inter-particle dimension (71). AlphaFold Server (53) was used to generate models of P-Rex1 DH/PH (38–409), P-Rex2 DH/PH (1–377), P-Rex1 DH/PH-DEP1 (38–499) and P-Rex2 DH/PH-DEP1 (1–467). The resulting models were used with their corresponding intensity profiles in BilboMD (38) to determine the model fit to the intensity profile and potential conformational heterogeneity.

## Data availability

The P-Rex2 EM data can be accessed as EMDB entries EMD-74547 (consensus map), EMD-74548 (local refinement of the N-terminal module), EMD-74549 (local refinement of the whole particle), EMD-74550 (composite map) and EMPIAR entry EMPIAR-13635. The P-Rex2 model has been deposited as PDB entry 9ZQ7. The SAXS data for P-Rex2 DH/PH and DH/PH-DEP1 can be accessed as SASDB entry SASDZV5 and SASDZU5, respectively.

## Supporting information

This article contains supporting information (25, 27, 36).

## Conflict of interest

The authors declare that they have no conflicts of interest with the contents of this article.
