# Supplementary material for: The Rho guanine-nucleotide exchange factor P-Rex2 exhibits structural and regulatory features distinct from the related RhoGEF P-Rex1
Source: J Biol Chem. 2026 Jun 4;302(7):113229. doi: 10.1016/j.jbc.2026.113229 (PMC13355178; doi:10.1016/j.jbc.2026.113229)
Supplement: Supporting Figures [file mmc1.pdf]

**The Rho guanine-nucleotide exchange factor P-Rex2 exhibits structural and regulatory features distinct from the related RhoGEF P-Rex1**

**Authors**

Lauren K. Anderson, Rohan Marde, Grace Muma, Veda Nayak, Chi Phan, Sheng Li, and Jennifer N. Cash

**Contents**

Supporting Information Figures S1-S11

**Figure S1. P-Rex2 cross-linking mass spectrometry (XL-MS) data agree with the cryo-EM structure of P-Rex2**
**Figure S2. P-Rex2 cryo-EM sample quality**
**Figure S3. Overview of P-Rex2 cryo-EM data processing pathway**
**Figure S4. Resolution and quality of P-Rex2 cryo-EM maps used for model building**
**Figure S5. Interpretability of maps and fit of P-Rex2 model**
**Figure S6. 2D class averages show that $IP_4$ binding does not stabilize a PH–IP4P interaction to allow resolution of the P-Rex2 IP4P subdomain**
**Figure S7. P-Rex2 SEC-SAXS data analysis**
**Figure S8. BilboMD re-analysis of P-Rex1 DH/PH and DH/PH-DEP1 SEC-SAXS data**
**Figure S9. SDS-PAGE of P-Rex2 DH/PH-DEP1 and DH/PH proteins used in Figure 6 GEF activity assays**
**Figure S10. Comparison of the αH/αI interface between P-Rex1 and P-Rex2**
**Figure S11. HDX-MS data on P-Rex2 with and without $IP_4$**

A

| P-Rex1 Homologous Residue 1 | P-Rex1 Homologous Residue 2 | Distance (Å) | P-Rex2 Residue 1 | P-Rex2 Residue 2 | Distance (Å) |
|---|---|---|---|---|---|
| 141 | 938 | 97.7 | 115 | 904 | Not modeled, unlikely to be within cross-linking distance |
| 154 | 1272 | 61.9 | 128 | 1212 | Unknown with current model |
| 241 | 1272 | 48.3 | 215 | 1212 | Unknown with current model |
| 164 | 1502 | 32.6 | 138 | 1442 | Not modeled, likely to be within cross-linking distance |
| 395 | 1502 | 22.2 | 364 | 1442 | 35.8 |
| 368 | 1502 | 32.8 | 337 | 1442 | 56.5 |
| 418 | 513 | 33.7 | 387 | 481 | 20.8 |
| 415 | 1502 | 42.0 | 384 | 1442 | 15.9 |
| 418 | 1502 | 45.1 | 387 | 1442 | 17.0 |
| 429 | 1502 | 54.4 | 397 | 1442 | 29.9 |

B.

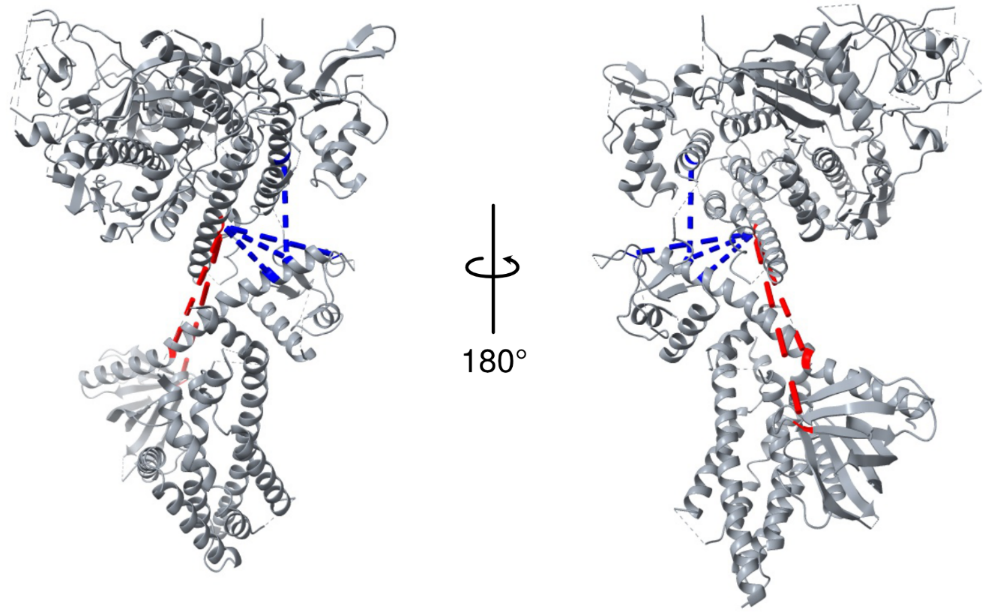


**Figure S1. P-Rex2 cross-linking mass spectrometry (XL-MS) data agree with the cryo-EM structure of P-Rex2.** A) Using P-Rex2 cross-linking mass spectrometry data from D'Andrea et al. (2021), we analyzed cross-links between residues in the N-terminal module and the C-terminal core. Looking at homologous residues in P-Rex1, we calculated the distances between Cα atoms of cross-linked residues using the structure of autoinhibited P-Rex1 (PDB: 8TUA). After obtaining the cryo-EM structure of P-Rex2, we performed the same calculations. B) P-Rex2 XL-MS data for cross-links between residues in the N-terminal module and the C-terminal core shown plotted onto the P-Rex2 structure. Blue dashes represent cross-linked residues that are <30 Å apart and red dashes represent cross-linked residues that are > 30 Å apart.

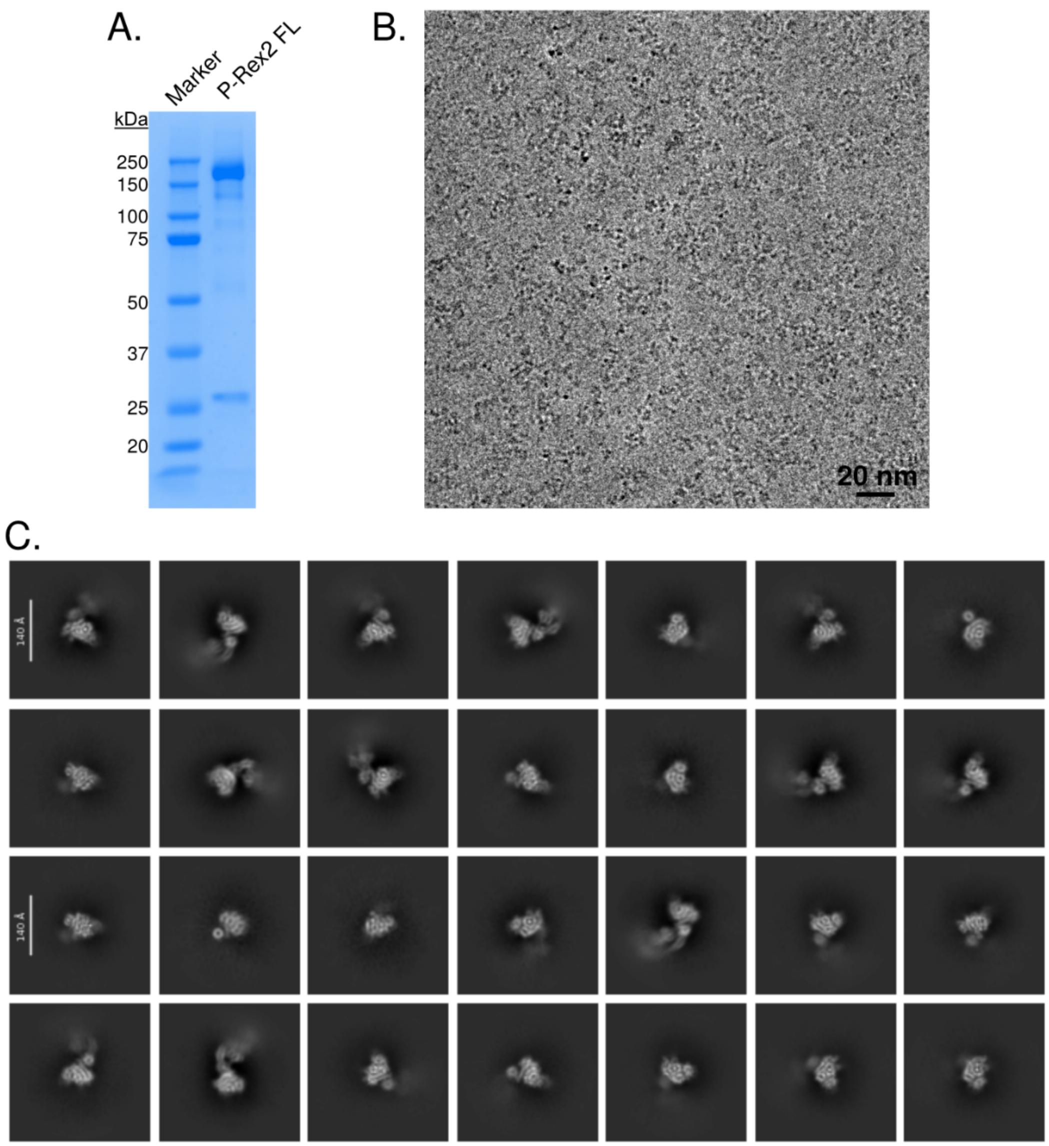


**Figure S2. P-Rex2 cryo-EM sample quality.** A) SDS-PAGE of purified P-Rex2 FL. B) Representative micrograph. C) Best 2D class averages from P-Rex2 data processing.

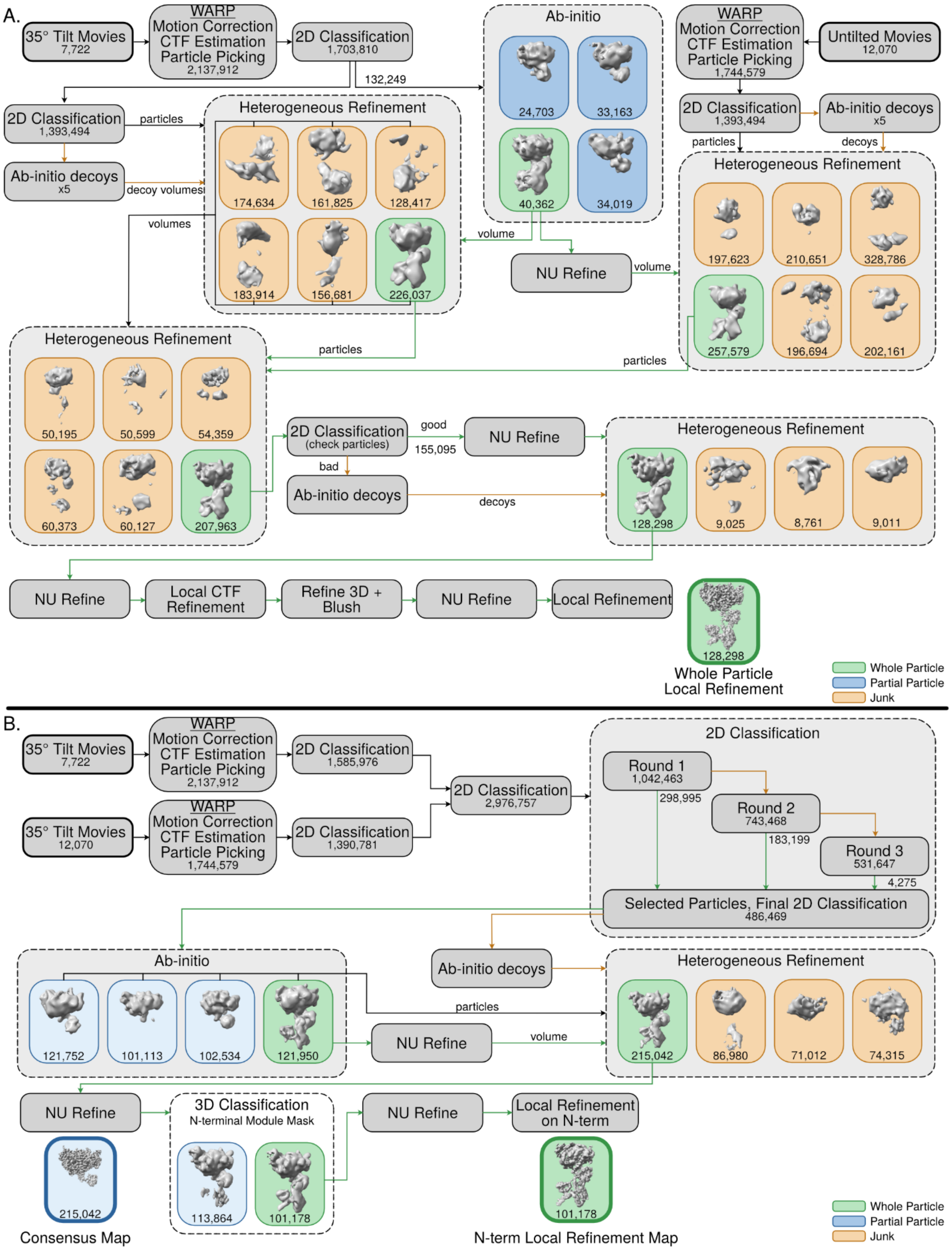


**Figure S3. Overview of P-Rex2 cryo-EM data processing pathway.** A) Data processing pathway of the whole particle local refinement map (EMD-74549). B) Data processing pathway resulting in the consensus map (EMD-74547) and N-terminal local refinement map (EMD-74548). Final maps from both pipelines are outlined with a thick line.

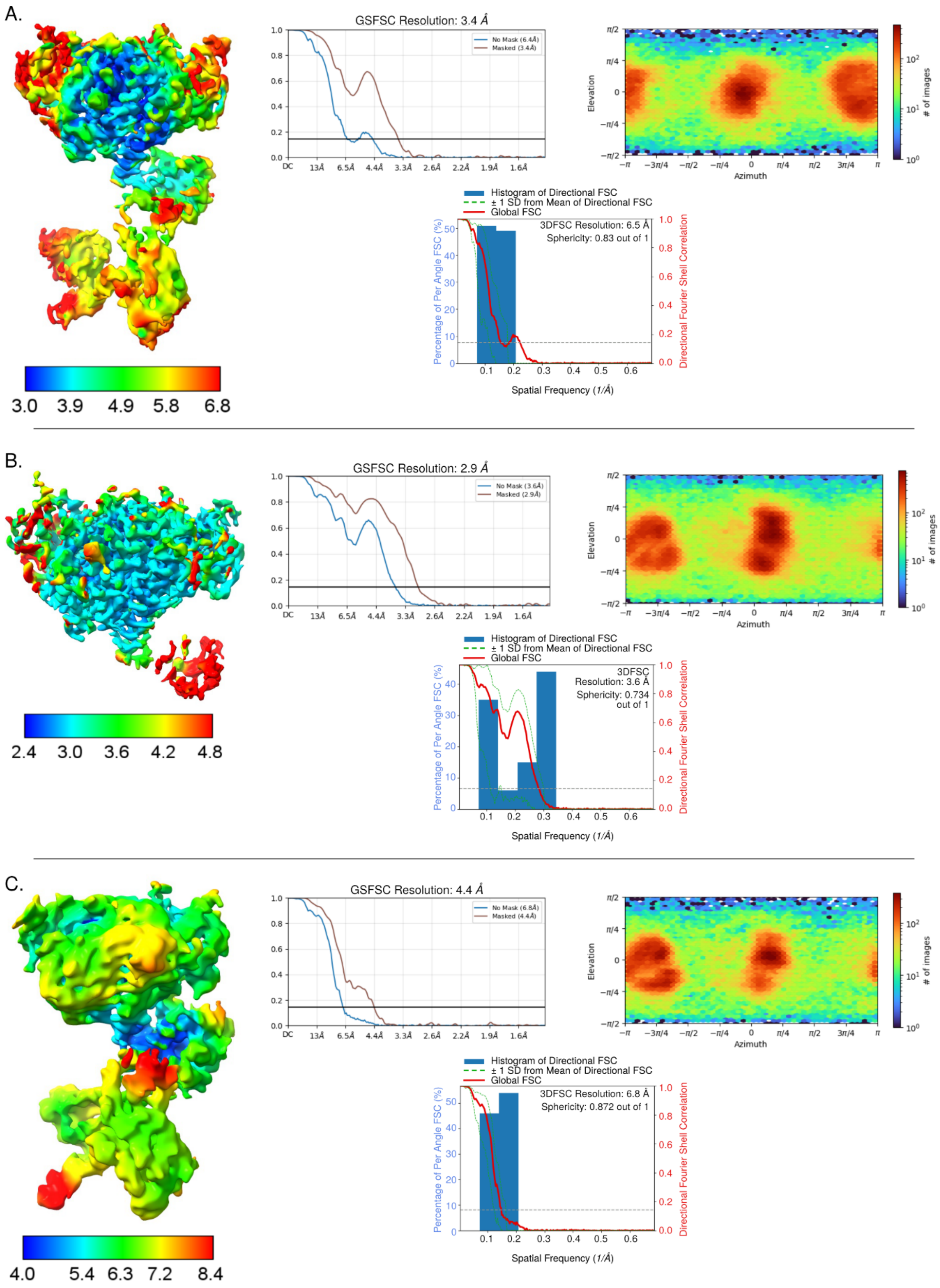


**Figure S4. Resolution and quality of P-Rex2 cryo-EM maps used for model building.** A) Map from local refinement of the whole particle, (B) consensus map, and (C) map from local refinement of the N-terminal module colored by local resolution, the corresponding cryoSPARC gold standard FSC curve and viewing direction distribution plot, and a 3D FSC histogram and directional FSC plot calculated by the 3D FSC program (https://3dfsc.salk.edu/).

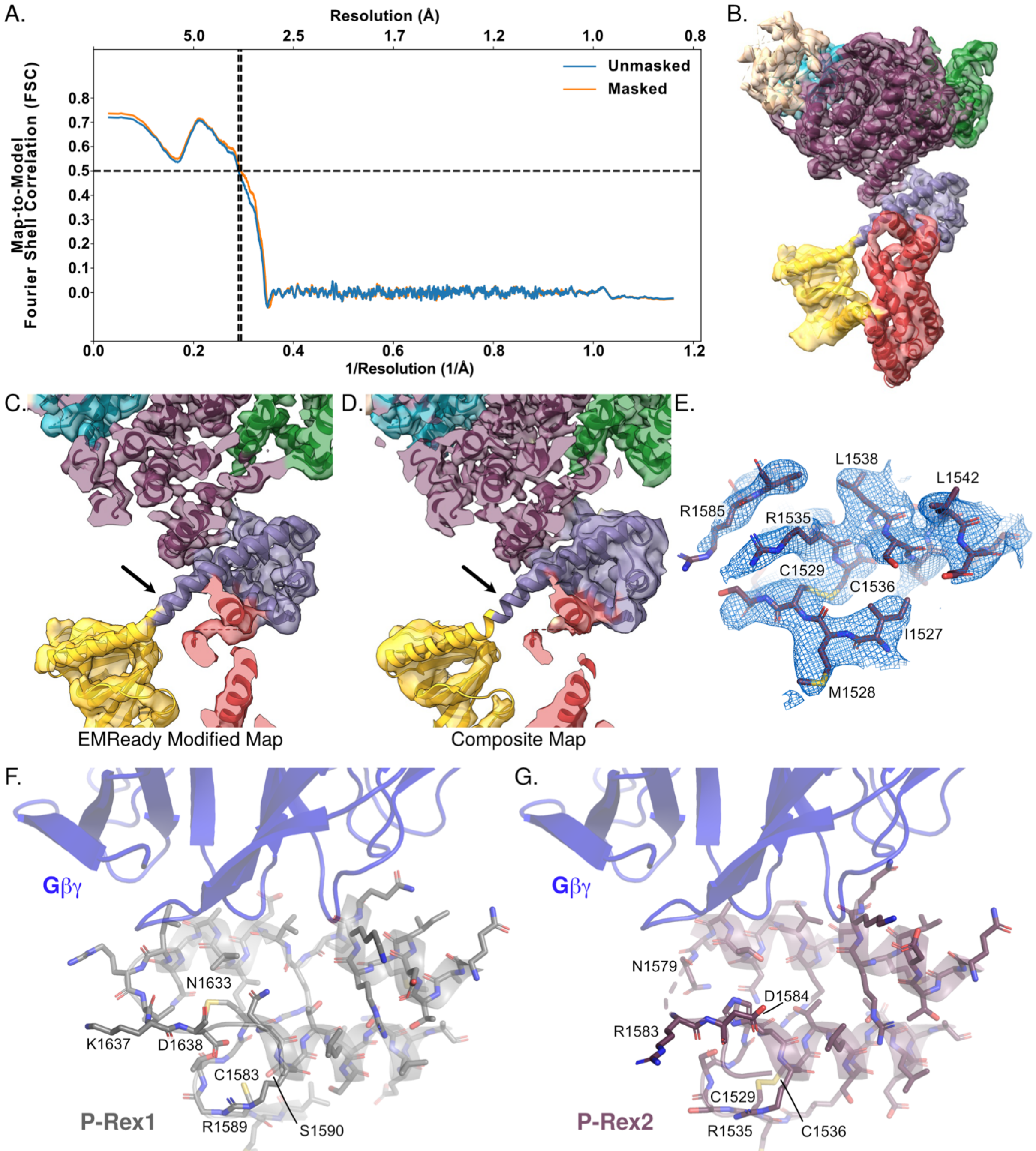


**Figure S5. Interpretability of maps and fit of P-Rex2 model.** A) Map-to-model FSC of the composite map. B) A density modified map generated with EMReady using the map from P-Rex2 whole particle local refinement. C-D) This map was especially useful in regions with poor resolution in the composite map such as in the helix bridging the PH and DEP1 domains (black arrow). E) P-Rex2 residues representing part of the "catalytic triad" (C1529, R1535, and D1584) of the proposed pseudo-phosphatase site and composite map density representing them, showing that this area is well-resolved in the map. Also shown here is the disulfide bond formed between C1529 of the triad and C1536, which is homologous to S1590 in P-Rex1. F) Part of the P-Rex1 Gβγ-binding site, also showing the triad (C1583, R1589, and D1638), with Gβγ shown semi-transparently (PDB: 6PCV). G) The equivalent site in P-Rex2 with Gβγ superimposed, showing differences in the triad as well as disorder in the loop upstream of D1584.

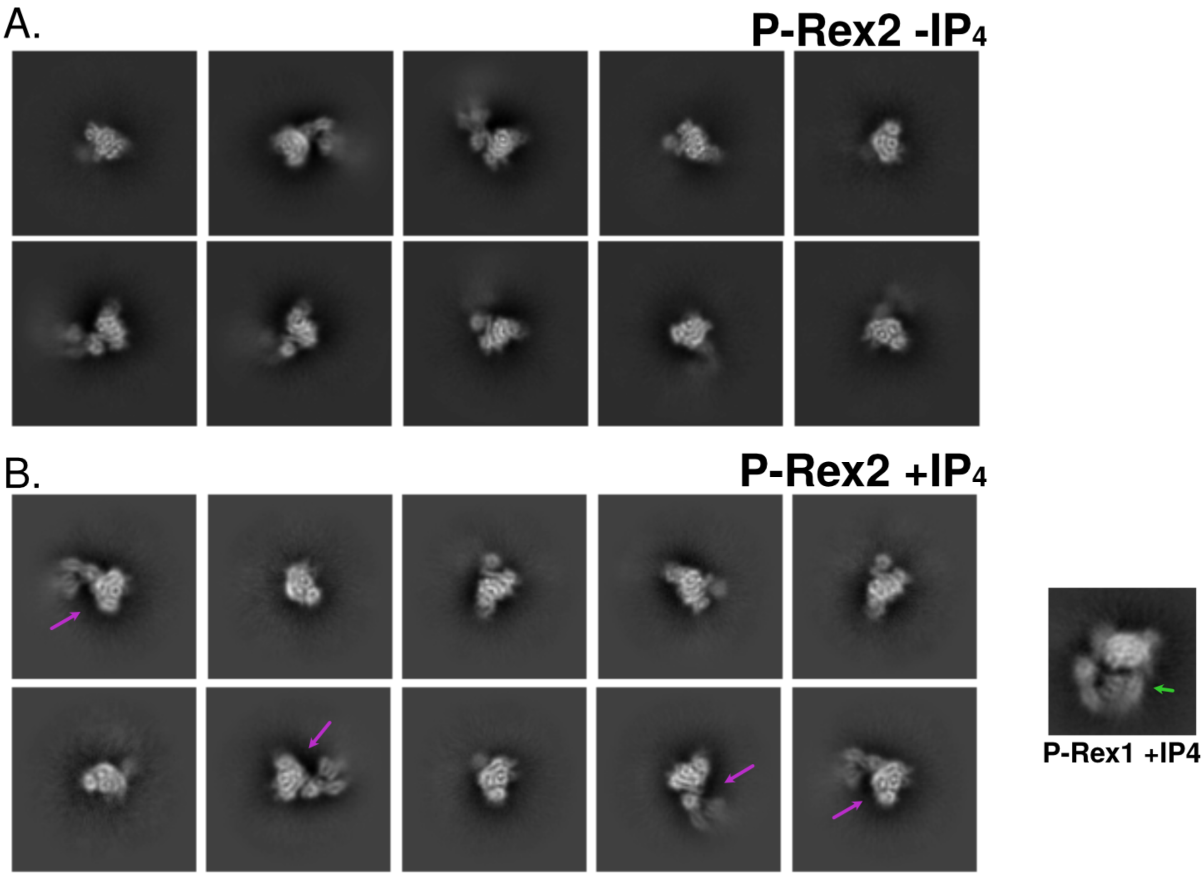


**Figure S6. 2D class averages show that $IP_4$ binding does not stabilize a PH–IP4P interaction to allow resolution of the P-Rex2 IP4P subdomain.** Two small datasets were collected on a Glacios on P-Rex2 samples in the A) absence and B) presence of $IP_4$. Datasets were processed in the same manner. A single 2D class average of P-Rex1 with $IP_4$ (Ravala et al., 2024) containing density for the IP4P subdomain (green arrow) is included to highlight its absence in P-Rex2 with $IP_4$ class averages (magenta arrow).

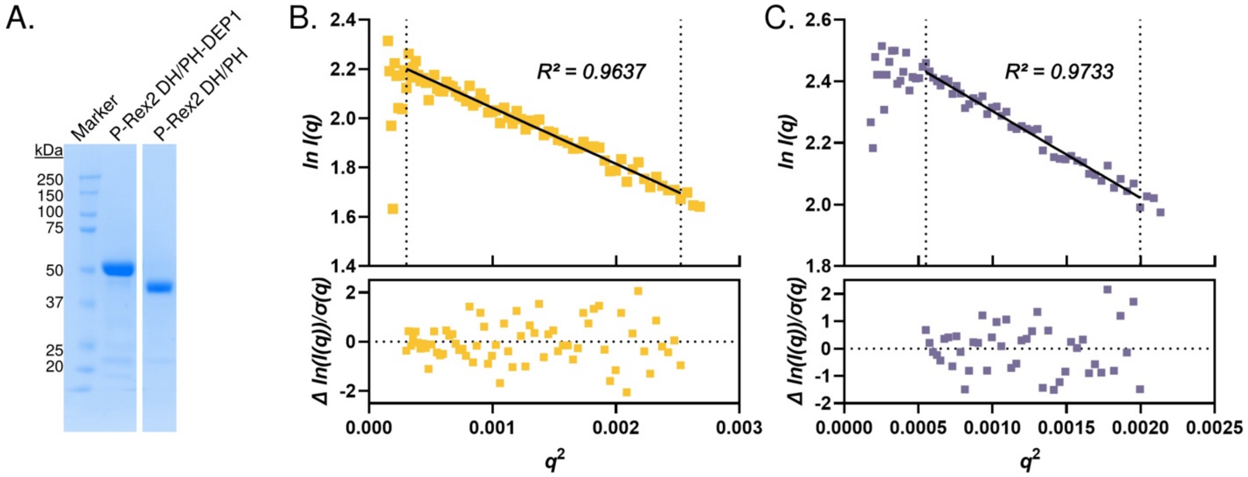


**Figure S7. P-Rex2 SEC-SAXS data analysis.** A) SDS-PAGE of purified P-Rex2 DH/PH and DH/PH-DEP1 constructs used in SEC-SAXS and GEF activity assay experiments. B) P-Rex2 DH/PH and C) P-Rex2 DH/PH-DEP1 Guinier plots.

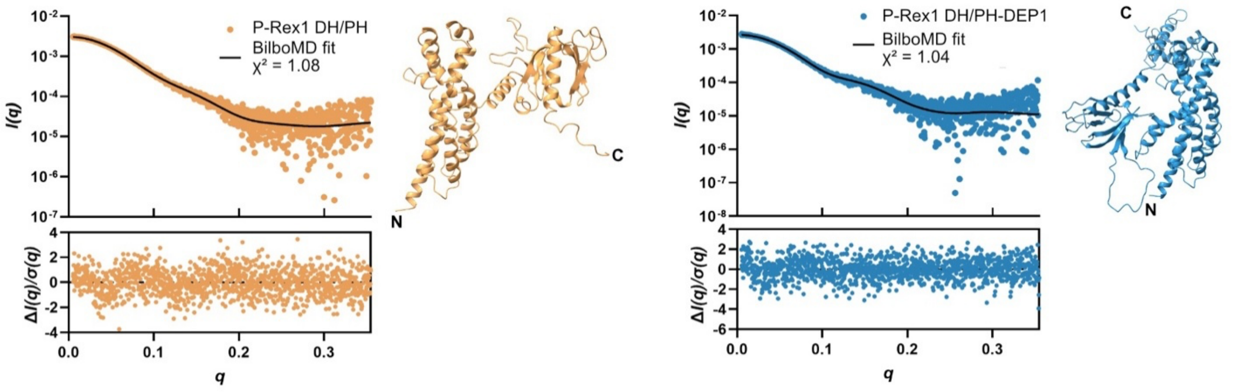


**Figure S8. BilboMD re-analysis of P-Rex1 DH/PH and DH/PH-DEP1 SEC-SAXS data.** Published SEC-SAXS datasets of P-Rex1 DH/PH and DH/PH-DEP1 (SASDHY9 and SASDHW9; Ravala et al., 2020) were analyzed using the BilboMD program. Scattering intensity plots of P-Rex1 DH/PH (orange) and DH/PH-DEP1 (blue) fit with the BilboMD model. The output BilboMD model is shown to the right of the plot. Normalized fit residuals are shown in the bottom panel.

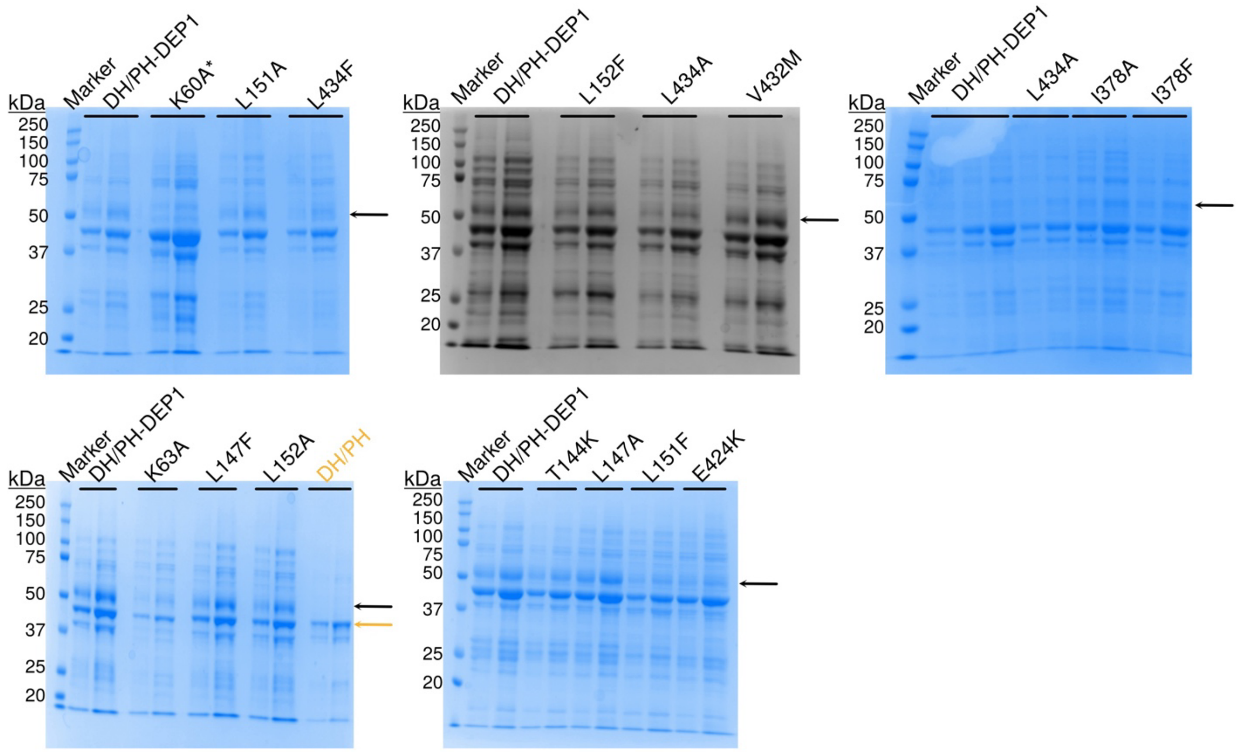


**Figure S9. SDS-PAGE of P-Rex2 DH/PH-DEP1 and DH/PH proteins used in Figure 6 GEF activity assays.** The black arrow indicates the band that represents each P-Rex2 DH/PH-DEP1 construct (~51 kDa). The yellow arrow indicates the band that represents P-Rex2 DH/PH (~44 kDa). For each construct, two lanes were run with different sample volumes to aid in estimating sample concentration.

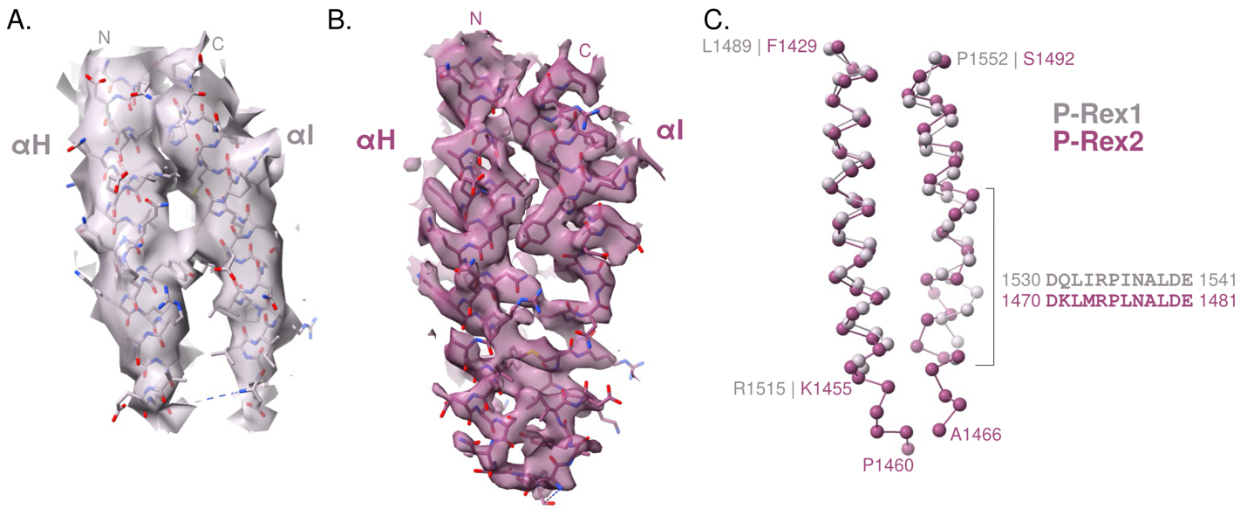


**Figure S10. Comparison of the αH/αI interface between P-Rex1 and P-Rex2.** Cryo-EM maps with the models fit showing how the map interpretability in regions around the αH and αI helices in A) P-Rex1 (PDB: 8TUA, EMDB-41621) and B) P-Rex2 are significantly different. There is also greater resolvability in the loop connecting these features in P-Rex2. C) The isolated Cα trace shows that this difference is most pronounced in the alternate structure from D1470 to E1481 in P-Rex2.

**Figure S11. HDX-MS data on P-Rex2 with and without $IP_4$.** Ribbon maps representing HDX-MS experiments with P-Rex2 alone and bound to $IP_4$. Also shown are the changes in exchange rates that occur in P-Rex2 upon $IP_4$ binding.

# Ribbon Map of P-Rex2 (% deuteration)

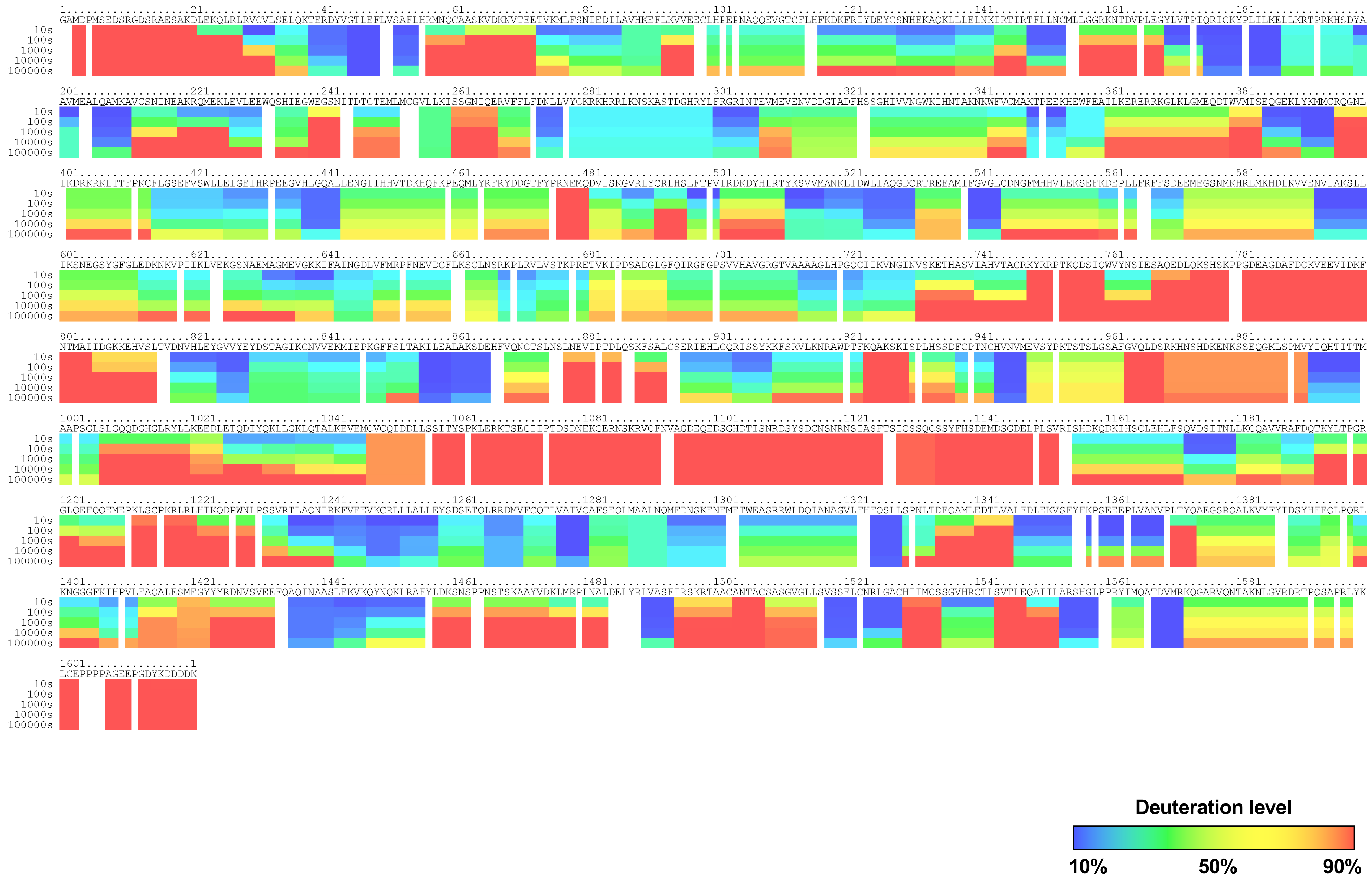

# Ribbon Map of P-Rex2 in P-Rex2•$IP_4$ Complex (% deuteration)

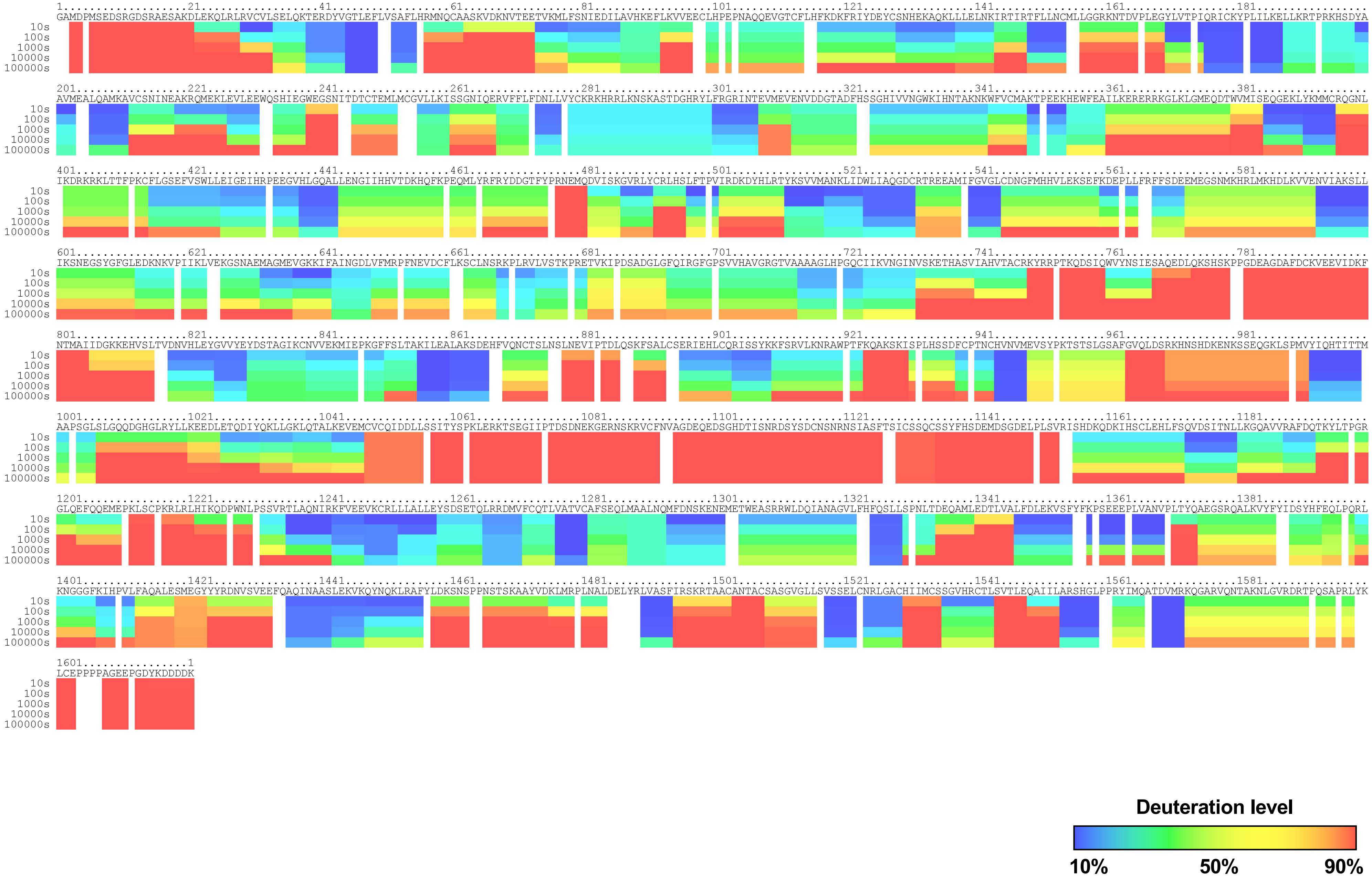

# Influence of $IP_4$ on Exchange in P-Rex2 (% deuteration)

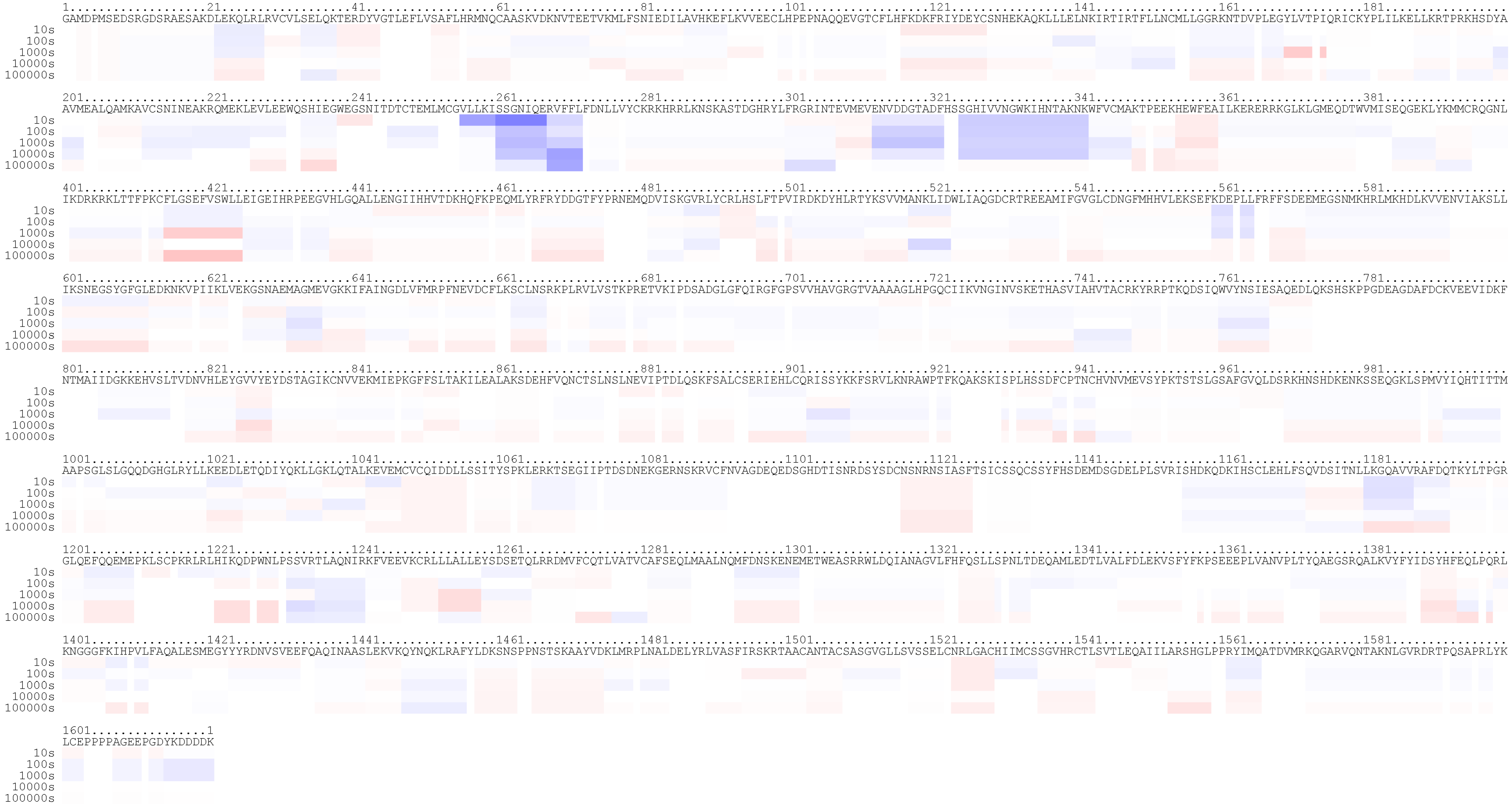

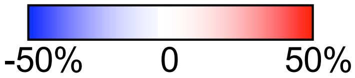


Blue indicates regions that exchange slower in the presence of $IP_4$.
Red indicates regions that exchange faster.
